# Open-MAC: A low-cost open-source motorized commutator for electro- and opto-physiological recordings in freely moving rodents

**DOI:** 10.1016/j.ohx.2023.e00429

**Published:** 2023-05-16

**Authors:** Sampath K.T. Kapanaiah, Dennis Kätzel

**Affiliations:** Institute of Applied Physiology, Ulm University, Ulm, Germany

**Keywords:** Motorized commutator, Active commutator, Swivel, In vivo, Open-ephys, Miniscope, SPI

## Abstract

In vivo electro- and optophysiology experiments in rodents reveal the neural mechanisms underlying behavior and brain disorders but mostly involve a cable connection between an implant in the animal and an external recording device. Standard tethers with thin cables or non-motorized commutators require constant monitoring and often manual interference to untwist the cable. Motorized commutators offer a solution, but those few that are commercially available are expensive and often not adapted to widely used connector standards of the open-source community like 12-channel SPI. Here we introduce an open-source motorized all-in-one commutator (Open-MAC): a low-cost (240–390 EUR), low-torque motorized commutator that can operate with minimal audible noise in a torque-based mode relying on dual magnetic Hall sensors. It further includes electronics to operate in a torque-free, online pose-estimation-based mode, with future developments. Operation is controlled by an onboard microcontroller (XIAO SAMD21) powered by a USB-C cable or DC power supply. The body and movable parts are 3D-printed. Different Open-MAC versions can support electrophysiology with up to 64 recording channels using the Open-Ephys / Intan^TM^ recording systems as well as miniature endoscope (miniscope) recordings using the UCLA Miniscope v3/4, and can host a fibre for optogenetic modulation.

## Graphical abstract

**Figure:**
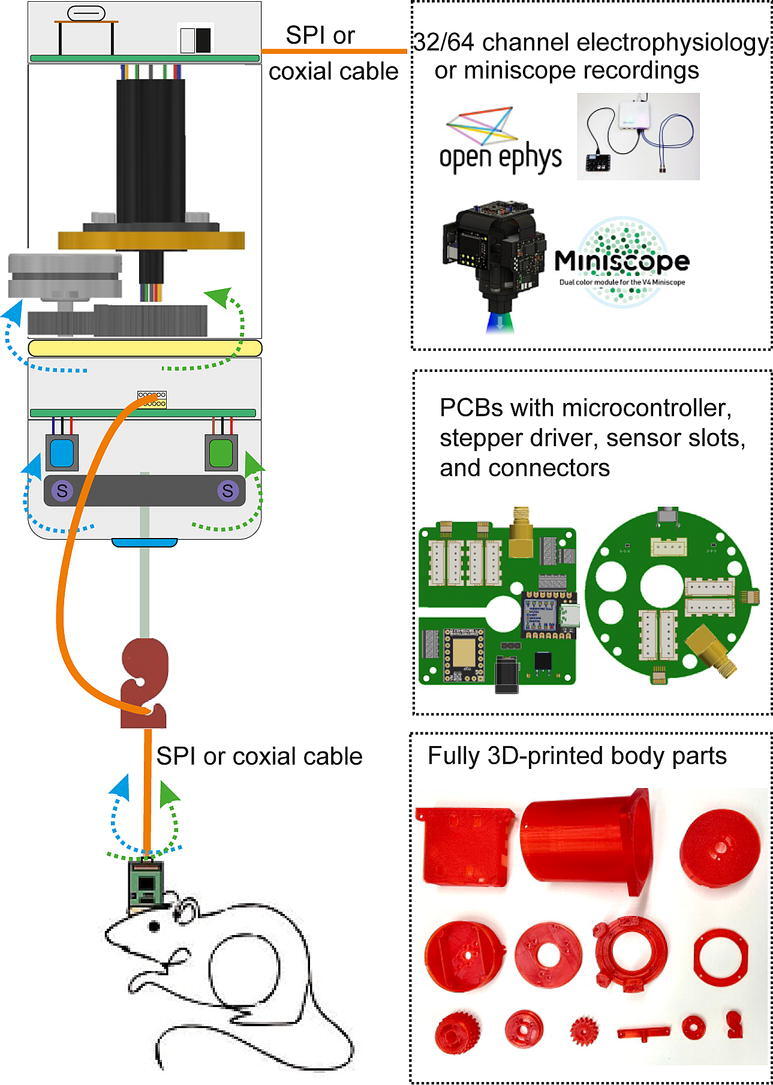

## Hardware in context

Open-source recording systems like the Open-Ephys project (https://open-ephys.org/) – based on Intan^TM^ technology (https://intantech.com/) – or the UCLA miniscope project [1] have provided the neuroscience community with high-quality, low-cost equipment to record a large number of channels or cells, respectively, in freely behaving rodents. These systems are widely used; for example Intan Technologies^TM^ sells well over a thousand headstage units per year (personal communication). However, such widely used equipment lacks a suitable and reliable low-cost, open-source commutator to enable investigators to record signals in a “walk-away-safe” setting. Instead, thin and flexible cables are often used that support several rounds of twisting as the animal makes turns. This approach can – however – be problematic in longer recordings or with more active animals in addition to risking bite-damages to the expensive cables. It also constitutes an unstable recording setting that requires constant monitoring and occasional manual interventions by the experimenter to untwist the cable when it winds up close to the animal’s head, which constitutes an unwanted disturbance of the experiment. Several commercial non-motorized commutators are available but are usually of limited use, as the very little torque exerted *on* and *through* such flexible light-weight cables by the animals is insufficient to turn the commutator.

Motorized commutators offer a solution but are hardly available commercially. A specialized motorized commutator from NeuroTek^TM^ is suitable for recordings with 12-channel *Serial Peripheral Interface* (SPI) cables via an Omnetics^TM^ PZN-12-AA connector (as are used by Open-Ephys/ Intan^TM^), but it is very expensive (>3500 USD) and has a broad body and fragile shape that cannot be easily attached above a maze or similar behavioural apparatuses (https://www.neurotek.ca/12–24-channel-motorized-commutator-for-intan-chips/). Two open-source solutions have recently been published [2], [3], but they are not engineered to support Open-Ephys-based recordings, i.e. to use 12-channel SPI-cables as input and output ports. An open-source design for the latter application has been developed as well [4], but this is, in turn, not applicable to miniscope recordings and - like the other motorized commutators stated above – it is not very compact.

We here developed a versatile motorized commutator that has multiple advantages. It combines low cost (ca. 240–390 EUR, depending on the used Omnetics^TM^ connectors and slip-ring that make up the largest share of the costs), a fully open-source – and hence modifiable – design, and a slick compact shape that allows easy and versatile attachment above behavioural apparatuses, with high functional versatility at the level of both channels and operational modes (see below). Although in principle applicable for other recording systems, it primarily represents a relatively easily implementable, robust, plug’n play motorized commutator for the large user community of the Open-EPhys/Intan^TM^ electrophysiology system and of the UCLA miniscope. For the former, it allows transmission of up to 64 recording channels (2 × 12 SPI channels), and for the latter it provides a co-axial transmission line for high-fidelity communication of high-frequency signals. Both features can be implemented in a single, versatile commutator or into separate, cheaper but purpose-built versions.

## Hardware description

### Design principles of the motorized commutator

#### General design

**Fig. 1:**
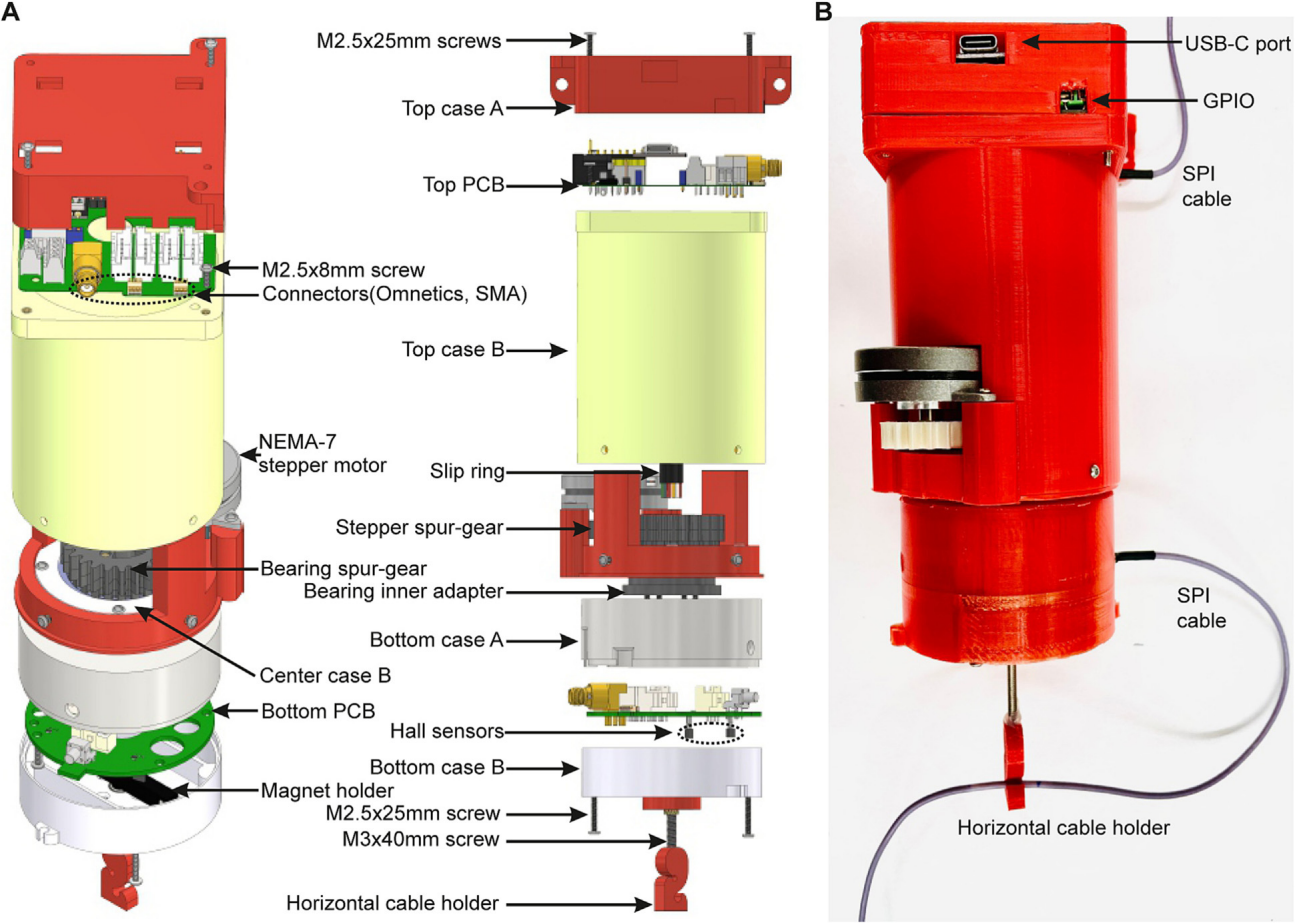
Schematic of Open-MAC. (A) Exploded view of the commutator in different horizontal perspectives with main parts annotated. (B) Side-view of fully assembled commutator.

The shape of the commutator is dominated by a slim, compact design to allow an easy and vertical fitting onto horizontal or vertical poles or grids above any behavioural arena or operant box (Fig. 1A–B). Multiple holes in the roof and side wall of the top case as well as the square shape above the main round body provide various options for attachment. The body case and all other plastic components, including spur-gears, are 3D-printed to reduce costs. Motorization is achieved via a NEMA14 stepper motor and silent stepper driver, reducing audible noise. We had developed and tested several earlier prototypes using geared DC motors (gear type N20) or a geared 2-phase mini stepper motor, but settled on the current design due to its superiority in terms of high torque, low audible noise and simplicity of assembly. Signal transmission through a rotating joint is achieved through a low-cost slip-ring at the center of the commutator; the type of incorporated slip-ring is determined by the required channels (see below; Fig. 1A–B).

**Fig. 2:**
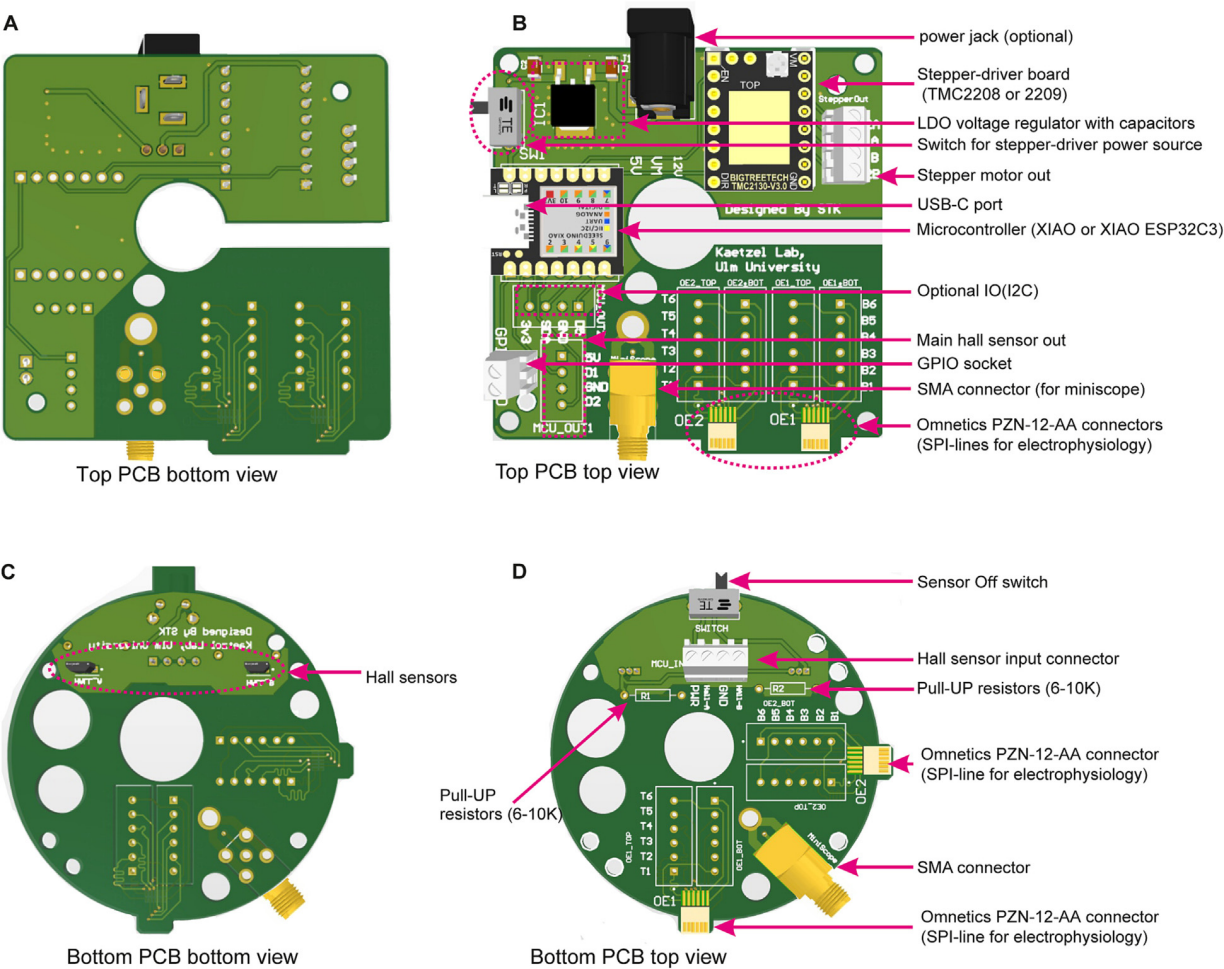
Overview of top and bottom PCB. (A-B) Top PCB shown from the bottom (A) and from the top (B). (B) shows almost fully assembled PCB with DC power jack barrel, 3-pin header, stepper-motor driver, XIAO microcontroller, one SMA and two Omnectics connectors added. (C-D) Bottom PCB shown from the bottom (C) and from the top (D). The images show almost fully assembled PCB with Hall sensors (C) as well as Hall sensor input connector, one SMA and two Omnectics connectors added (D).

One printed circuit board (PCB) each at the top and bottom incorporates all necessary electronic functionality including the connector boards for the input and output data cables (Fig. 1A, 2A–D). Additionally, the bottom PCB incorporates two magnetic Hall sensors to allow for the sensing of torque for operation in the standard, torque-based mode (Fig. 2C–D, 3A). The top PCB controls the operation of the commutator through a stepper-motor driver board and incorporates three further ports (Fig. 2A–B):

(1) a *multi-purpose USB-C port* that can be used to power the commutator (including its motor), to program its microcontroller (MCU) setting operational parameters with Arduino IDE (code provided with this manuscript) or CircuitPython, and to provide information about the animal’s rotation in the torque-free operational mode, when developed in future (see below).
(2) a *general purpose input/output (GPIO) port* which can be used to generate transistor-transistor logic (TTL) signals whenever the commutator’s motor is turning to time-stamp physiological recordings with this information, or to receive tracking information in the torque-free operational mode.
(3) a DC jack barrel to power the commutator via a 12 V power adapter in case the USB-C port is not used for this purpose. Note that the USB-C cable, connected to the acquisition computer, should be preferred as power supply to avoid 50 Hz or 60 Hz noise that could be imposed by an external power supply.

**Fig. 3:**
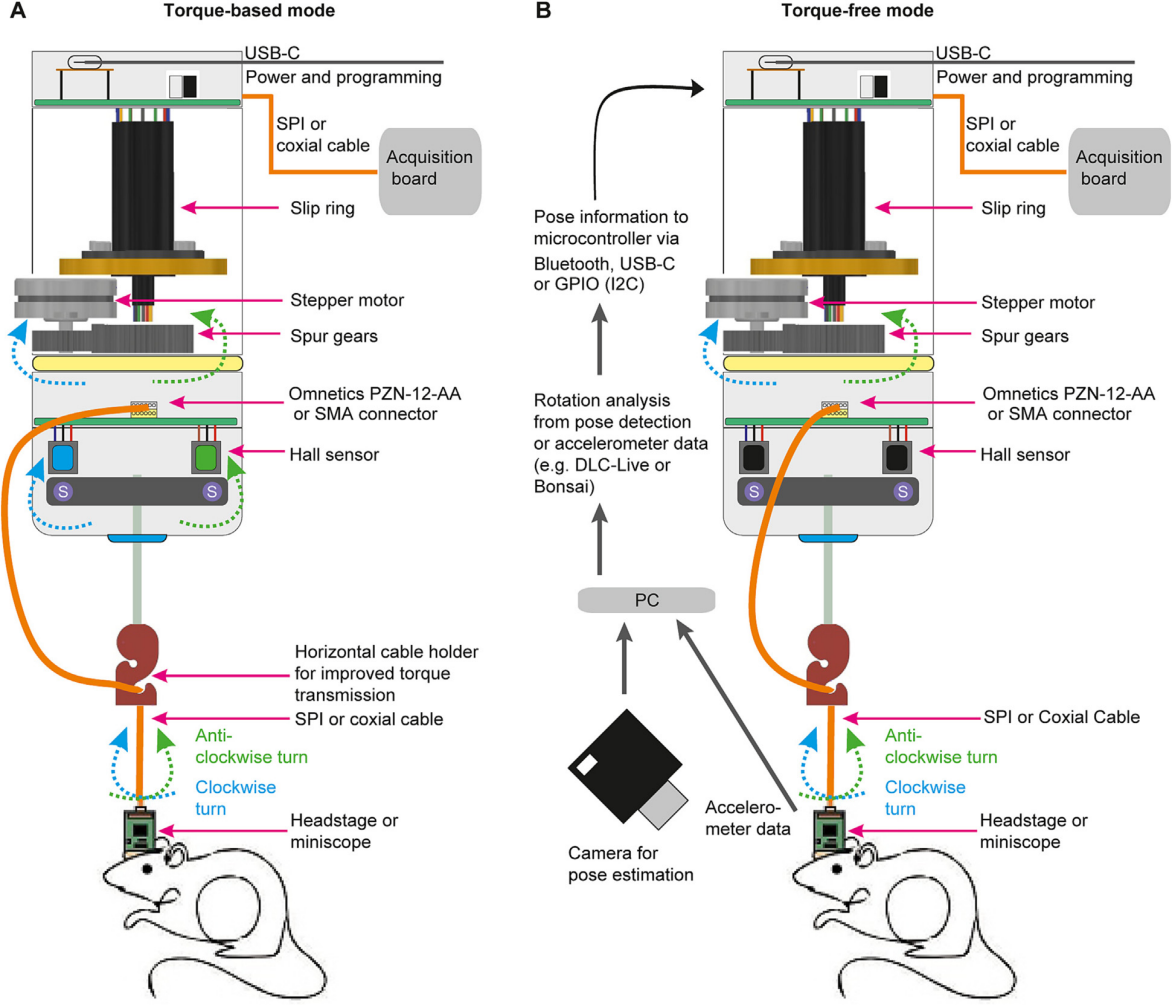
Two modes of operating Open-MAC. (A) Illustration of torque-based mode in which the activation of the motor is determined by the torque exerted onto the cable by the animal’s rotation. (B) Torque-free mode in which the activation of the motor is determined by the measurement of the animal’s rotation from either accelerometer data or from pose estimation.

#### Versatility of channel usage

The commutator has primarily been designed to be directly applicable for Open-EPhys / Intan^TM^ systems by providing singular 12-channel SPI connectors (Omnetics^TM^ PZN-12-AA polarized nano, part number A79623-001) as input and output (Fig. 1, Fig. 2) enabling the recording of 32 analogue measurement channels plus duplicates of ground and reference (36 electrophysiological channels in total) in addition to 3 accelerometer channels. The printed circuit boards (PCBs) of the input and output stage can, however, accommodate another SPI-connector each (Fig. 2), thereby duplicating the available number of electrophysiological measurement channels to 64. A third port on the PCBs enables high-fidelity, high-speed dual-channel (signal-ground) communication in the radio-frequency (RF) range through an SMA-port, and therefore makes the commutator suitable for optophysiological recordings with miniature endoscopic microscopes (miniscopes; Fig. 1, Fig. 2). Finally, an optional through-hole in the middle may enable an additional application of optical fibres to support, for example, optogenetic modulation in parallel with physiological recordings. The actual versatility of each exemplar of this commutator is dependent on the input- and output-connectors that are actually added to it and the corresponding slip-ring incorporated into its body; for example, one commutator could be built only with SMA-connectors for miniscope-recordings, while another could be equipped solely with SPI-connectors for use with electrophysiology, and a third one could feature both connectors so that it can be employed in both types of experiments.

We deliberately developed a range of commutators, suitable for specific purposes, rather than a single one that incorporates maximum channel count and all options combined. This is because the latter strategy would come at comparably high cost per commutator, as the required SPI connectors and slip-ring are the main cost drivers, by far. Instead we suggest to rather build many cheaper, purpose-specific commutators and benefit from the simultaneous experimentation that the walk-away safety, it allows, provides. Nevertheless, our design can be used with few adaptations to generate even more versatile commutators than the combined 12-channel SPI (electrophysiology)/1-channel coaxial (miniscope) or 12-channel SPI (electrophysiology)/optic-fibre (optogenetic) versions, described here, represent. Specifically, it is also possible to build a 24-channel SPI plus optic-fibre through-hole commutator by slightly modifying just the slip-ring stage of our design, as well as a 24-channel SPI plus co-axial (miniscope) commutator when incorporating another slip-ring and accommodating it by changes to multiple parts of the body. Notably, it is not easily possible to adapt our design to combine a coaxial channel (miniscope) with a fibre-through hole since Senring^TM^ does not offer a suitable slip-ring for this design.

#### Operational modes of the motorized commutator

The commutator can theoretically be used in two different modes; in the *torque-based mode*, in which a turning of the lower cable by the moving animal is detected by Hall sensors (Fig. 3A), and the *torque-free mode* (to be fully developed in future), in which the turning of the animal is determined by online-tracking, e.g. using DeepLabCut-live (DLClive) [5] or DeepLabStream [6], or from accelerometer data (Fig. 3B). In either case, a signal is generated that is translated into the activation of the stepper-motor to compensate the turning of the animal by twisting the cable by a corresponding amount (Fig. 3). In the *torque-free mode*, this signal is transmitted via a Bluetooth sensor implemented in the upper PCB, but it can also be sent through its GPIO or its USB-C port. This mode would allow to counteract the animal’s rotations with high spatial and temporal precision and may provide advantages for users that use an online pose-estimation system already, particularly in cases where the cable is long (e.g. for larger arenas) and/or very flexible, limiting the effective transmission of rotation-induced torque.

Note that, at the current stage of application and testing, the *torque-based mode* is what we have comprehensively developed, tested and used as it is the default and simplest operational mode requiring only the commutator itself, as described here, functioning as a stand-alone device. The *torque-free mode* has been enabled by our design at the level of hardware as a measure of future-proofing and opportunity for further applications, but we have not actually used and tested it ourselves at this stage, as it requires additional implementations at the software-level via an external computer (such as the acquisition PC of the set-up) (Table 1).

**Table 1:** Specifications table.

**Hardware name**	*Open-MAC (open-source motorized all-in-one commutator)*
**Subject area**	*Neuroscience*
**Hardware type**	*Electro- and optophysiological measurement (commutator)*
**Closest commercial analog**	*There is no commercial analogue for a motorized commutator that supports both of the described electro- and optophysiological recordings. The closest commercial analogue for the main application (electrophysiology) would be the product CMTR-12*–*24-M−INTAN−NT from Neurotek, CA (**https://www.neurotek.ca/12–24-channel-motorized-commutator-for-intan-chips/**)*
**Open source license**	*GNU General Public License (GPL) 3.0 and later* for Hardware and Software
**Cost of hardware**	*240*–*390 EUR* *Note that the costs depend on the type commutator to be built (as we introduce different versions here, for distinct applications) because the largest part of the costs are the SPI-connectors and the slip-ring of which different numbers or types, respectively, are used depending on the type of commutator built*
**Source file repository**	https://zenodo.org/record/7640543 https://doi.org/10.5281/zenodo.7640543 https://github.com/KaetzelLab/Open-MAC
**OSHWA certification UID**	DE000134

## Design files summary

**Fig. 4:**
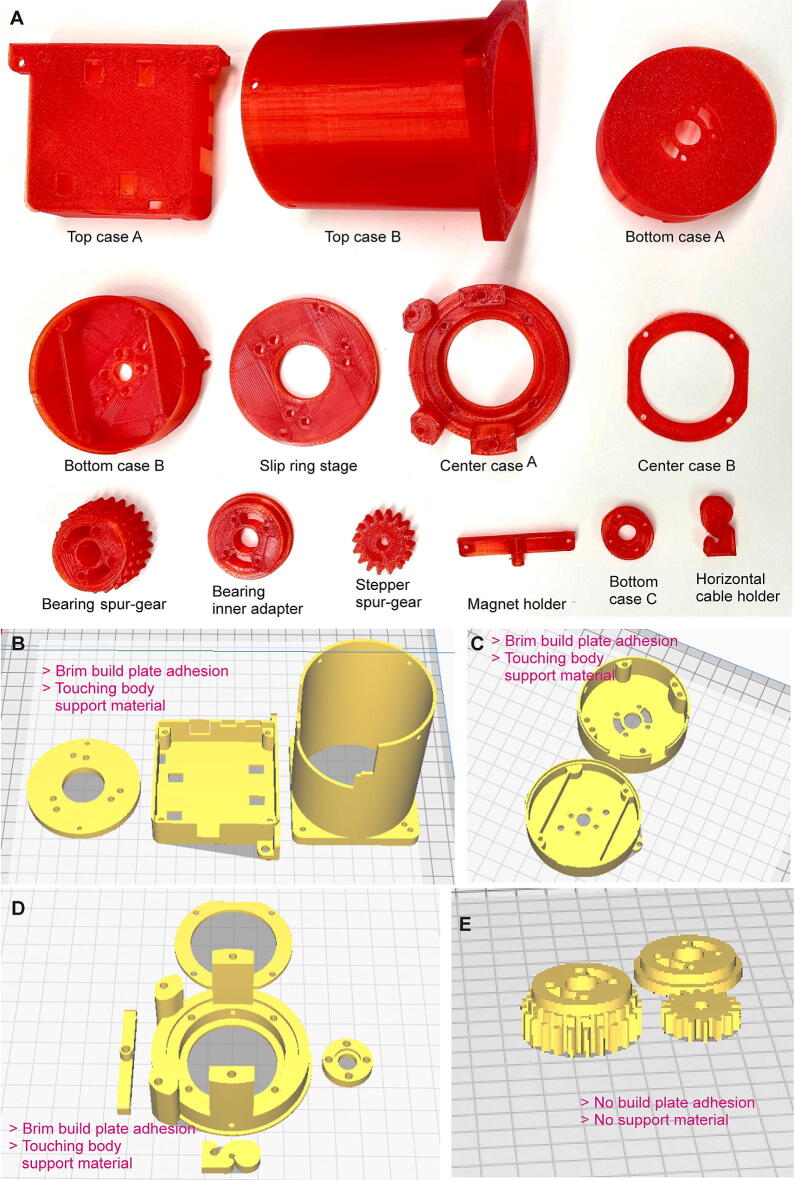
3D-printed parts of Open-MAC. (A) Overview of all 3D-printed parts used in the commutator, annotated as in Table 2 and Fig. 1. (B-E) 3D-models and their orientation in the 3D-Slicer software. Required printing parameters are stated in magenta. (For interpretation of the references to colour in this figure legend, the reader is referred to the web version of this article.)

Below is a short explanation of the *3D-printed elements* of the commutator, which are also shown in Fig. 4A–E and listed in Table 2:

**Table 2:** Design files. The two upper tables list the design files for all 3D-printed components of Open-MAC, which we provide once as stl-files for direct 3D-printing (top table) and once as f3d-files (middle table) that allow future modifications to the design. The bottom table lists the design files for the PCBs. All designs are covered by the *GNU General Public License (GPL) 2.0* and are supplied as zip-Folders on https://zenodo.org/record/7640543.

**Part name**	**Design filename**	**File Type**	**OS licence**	**Location of the file**
Top case A	1 Com Top A.stl	stl	GPL	https://zenodo.org/record/7640543
Top case B	2 Com Top B.stl	stl	GPL	https://zenodo.org/record/7640543
Slip ring stage	3 Com Slip Ring stage.stl	stl	GPL	https://zenodo.org/record/7640543
Center case A	4 Com Center case A.stl	stl	GPL	https://zenodo.org/record/7640543
Center case B	5 Com Center case B.stl	stl	GPL	https://zenodo.org/record/7640543
Bearing spur gear	6 Com Bearing Spur Gear.stl	stl	GPL	https://zenodo.org/record/7640543
Stepper spur gear	7 Com Stepper Spur Gear.stl	stl	GPL	https://zenodo.org/record/7640543
Bearing inner adapter	8 Com Bearing Inner Adapter.stl	stl	GPL	https://zenodo.org/record/7640543
Bottom case A	9 Com Bottom A.stl	stl	GPL	https://zenodo.org/record/7640543
Bottom case B	10 Com Bottom B.stl	stl	GPL	https://zenodo.org/record/7640543
Magnet holder	11 Com Magnet holder.stl	stl	GPL	https://zenodo.org/record/7640543
Bottom case C	12 Com Bottom C.stl	stl	GPL	https://zenodo.org/record/7640543
Horizontal cable holder	13 Com horizontal cable holder.stl	stl	GPL	https://zenodo.org/record/7640543
**Part name**	**Design filename**	**File Type**	**OS licence**	**Location of the file**
Top case A	Com Top A.f3d	f3d	GPL	https://zenodo.org/record/7640543
Top case B	Com Top B.f3d	f3d	GPL	https://zenodo.org/record/7640543
Slip ring stage	Com Slip Ring Stage.f3d	f3d	GPL	https://zenodo.org/record/7640543
Center case A	Com Center Bearing Assembly.f3d	f3d	GPL	https://zenodo.org/record/7640543
Center case B	Com Center Bearing Assembly.f3d	f3d	GPL	https://zenodo.org/record/7640543
Bearing spur gear	SpurGears.f3d	f3d	GPL	https://zenodo.org/record/7640543
Stepper spur gear	SpurGears.f3d	f3d	GPL	https://zenodo.org/record/7640543
Bearing inner adapter	SpurGears.f3d	f3d	GPL	https://zenodo.org/record/7640543
Bottom case A	Com bottom assembly.f3d	f3d	GPL	https://zenodo.org/record/7640543
Bottom case B	Com bottom assembly.f3d	f3d	GPL	https://zenodo.org/record/7640543
Magnet holder	Com Magnet holder.f3d	f3d	GPL	https://zenodo.org/record/7640543
Bottom case C	Com bottom assembly.f3d	f3d	GPL	https://zenodo.org/record/7640543
Horizontal cable holder	Com horizontal cable holder.f3d	f3d	GPL	https://zenodo.org/record/7640543
**Part name**	**Design filename**	**File Type**	**OS licence**	**Location of the file**
Top PCB	Top PCB.zip	gerber	GPL	https://zenodo.org/record/7640543
Bottom PCB	Bottom PCB.zip	gerber	GPL	https://zenodo.org/record/7640543

**Top case A:** Housing for top PCB, also helps to mount commutator.

**Top case B:** Cover of the slip-ring, attaching to top PCB, Top case A and Center case A assembly.

**Slip ring stage:** Stage to mount slip-ring.

**Center case A:** Center-connecting body, also holding the stepper motor, bearing assembly and slip-ring stage.

**Center case B:** Helps to secure ball-bearing in place.

**Bearing spur-gear:** Transfers torque from stepper spur-gear to bottom assembly.

**Stepper spur-gear:** Connected to shaft of the stepper motor and turns bearing spur-gear when shaft turns.

**Bearing inner adapter:** supporting structure between bottom case A and center assembly, also provides spacing between rotating bottom assembly and non-rotating top assembly.

**Bottom case A:** Connecting body to bearing inner adapter and bottom PCB.

**Bottom case B:** Secures bottom PCB and houses the magnet mount.

**Magnet holder:** Holds two small magnets on its sides; attached to M3 rotor.

**Bottom case C:** Secures small bearing onto bottom case B.

For 3D-printing we used Polylactic acid (PLA) filament with a 4 mm nozzle, a 0.2 mm layer height, a 65% infill density, and brim setting for build plate adhesion for most parts, except for the stepper spur-gear, bearing spur-gear and bearing inner adapter (Fig. 4B–E). We provide stl-files for 3D-printing as well as f3d-files to enable further design changes by the user (using Autodesk Fusion360) according to specific experimental requirements (Table 2). We opted for using an infill density of 65% (generally ≥ 50%) for these parts to enhance the stability of the case and of the moving gears.

**Fig. 5:**
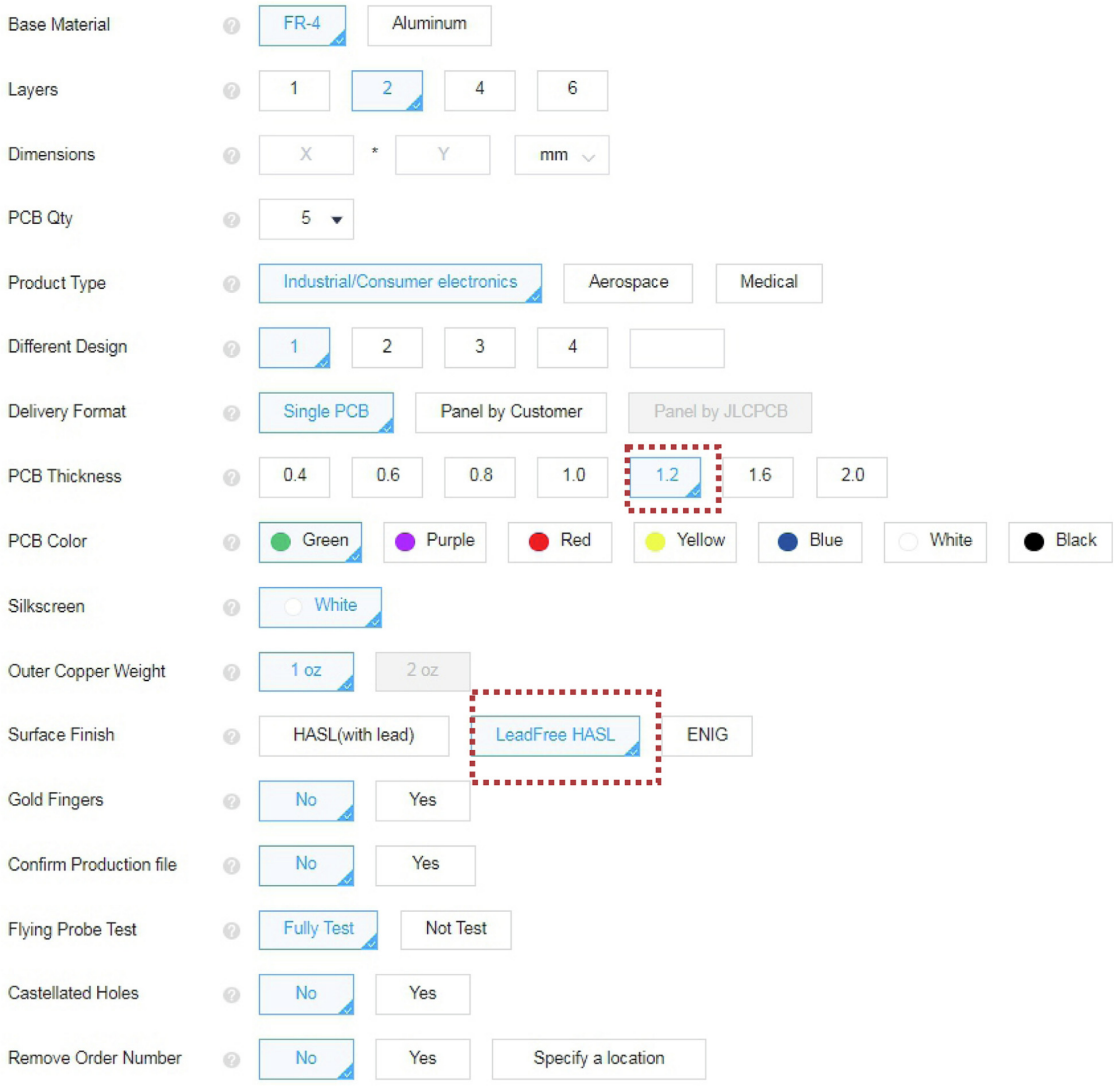
PCB manufacturing specifications. (A) Screen shot of the ordering process on the JLC PCB website (https://jlcpcb.com/). Chosen default parameters are in blue boxes. Important parameters are marked in red. (For interpretation of the references to colour in this figure legend, the reader is referred to the web version of this article.)

PCBs (Fig. 2, Table 2) can be and have been ordered from JLC PCB (https://jlcpcb.com/) by uploading the gerber zip files listed below. An alternative manufacturer is PCBWay (https://www.pcbway.com/). The thickness of the PCBs should be 1.2 mm while the remaining parameters can be left at the default value or can be change as per the user’s requirements (see Fig. 5 for ordering parameters of PCBs).

## Bill of materials summary

**Fig. 6:**
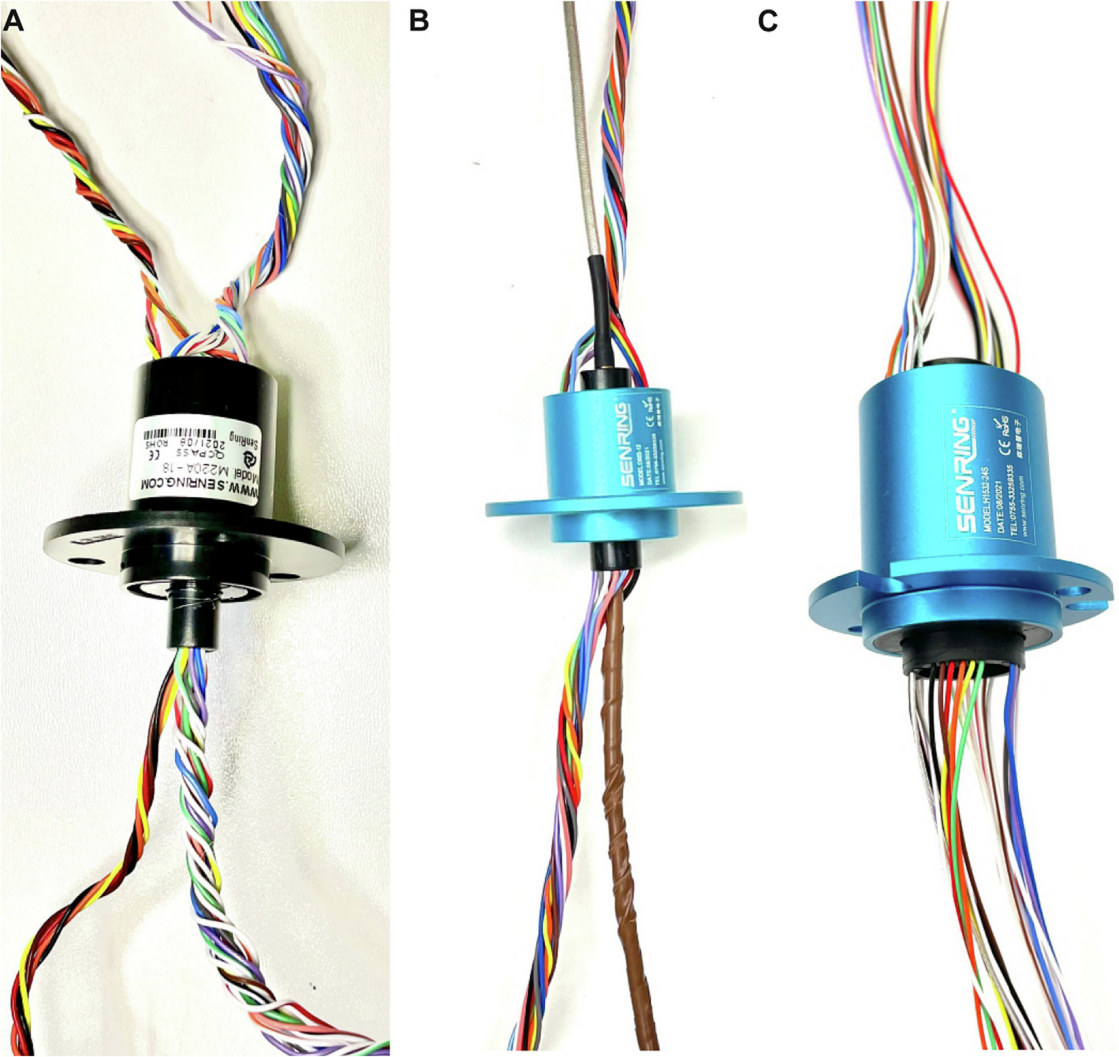
Slip-ring options for different applications. (A) Senring^TM^ M220-A to support a 12-channel SPI connection for electrophysiology applications only. (B) Senring^TM^ O022-12 with a high-speed RF line for miniscope recordings only. (C) Senring^TM^ H1532-24S for use with a central optical fibre for optogenetics and a 12-channel SPI connection for simultaneous electrophysiological recordings.

Fig. 6 shows three distinct slip-rings that are used for different versions of the commutator including electrophysiology only (A), miniscope recordings only (B), and electrophysiology in combination with optogenetic modulation (C). Note that the latter set-up also requires an optical commutator to permit simultaneous rotation of the optical fibre, as is commonly used in optogenetics.

## Build instructions

### Step 1: Preparing the slip-ring

**Fig. 7:**
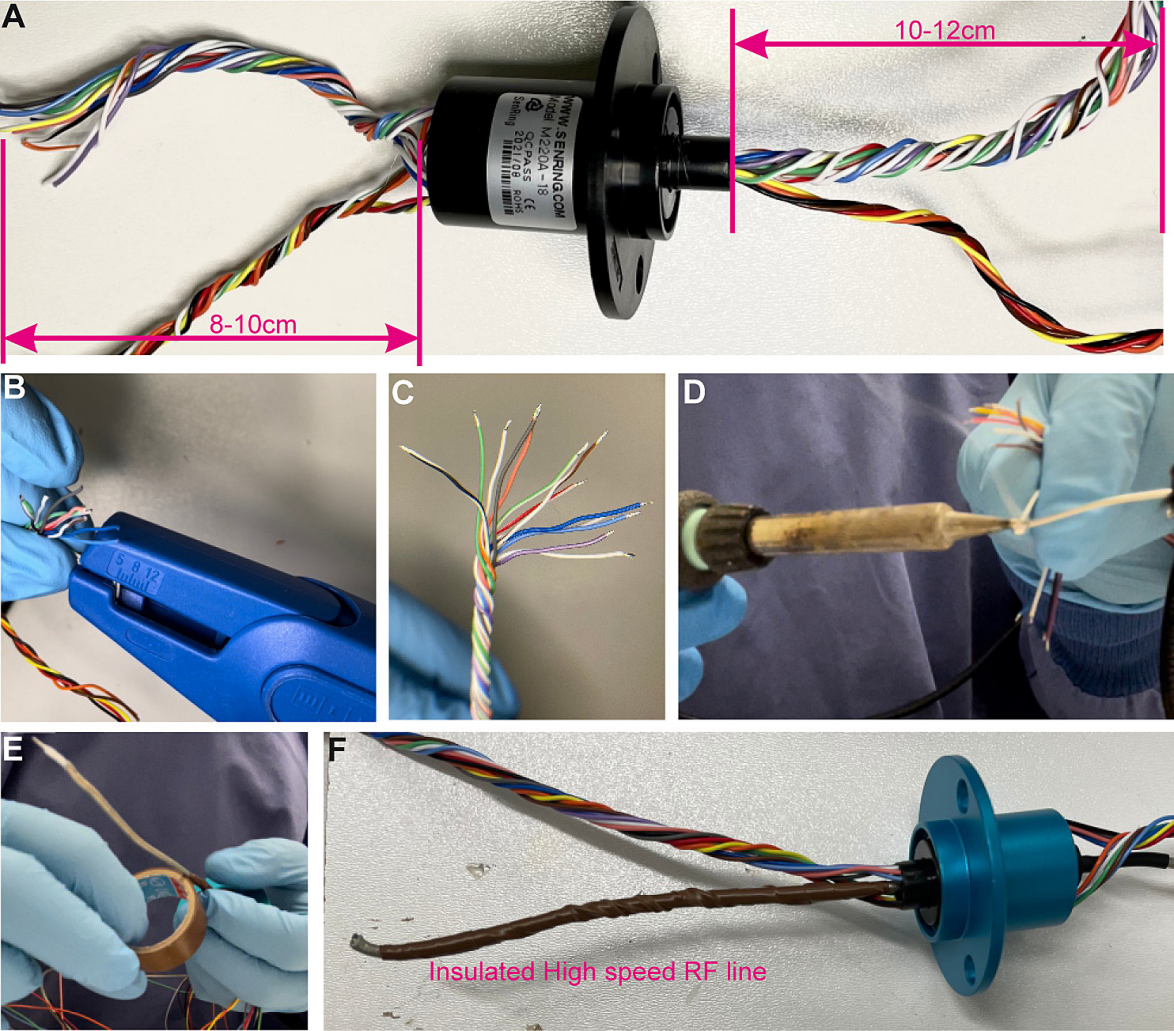
Slip-ring preparation. (A) Slip ring and required cable lengths. (B) Stripping of cables. (C) Appearance when all cables are properly stripped. (D) Application of solder to stripped cable ends (priming). (D-E) Covering insulating tape to high-speed RF line used for miniscope imaging (coloured cables above represent twisted SPI-lines in F).

Cut wires of slip-ring to same length of approx. 8–12 cm on both ends. Group and twist wires together according to their functions; i.e. the 12-SPI lines that carry the electrophysiological signal (including accelerometer signal, if present) are grouped together and the Hall sensor wires are grouped separately (Fig. 7A). Strip the insulation of the last 2 mm of all cable ends and prime stripped wire ends with fresh solder (Fig. 7B–D; images show the Senring^TM^ M220A-18 slip-ring used for electrophysiology-only commutators with a single 12-channel SPI port at both ends). In case the commutator should be used for miniscope imaging, requiring a high-speed radio-frequency (RF) transmission line, this cable needs to be insulated separately using insulation tape if not covered already by the manufacturer (Fig. 7E–F, showing Senring^TM^ slip-ring O022-12; see also Fig. 6A–B).

### Step 2: Preparing the top PCB

**Fig. 8:**
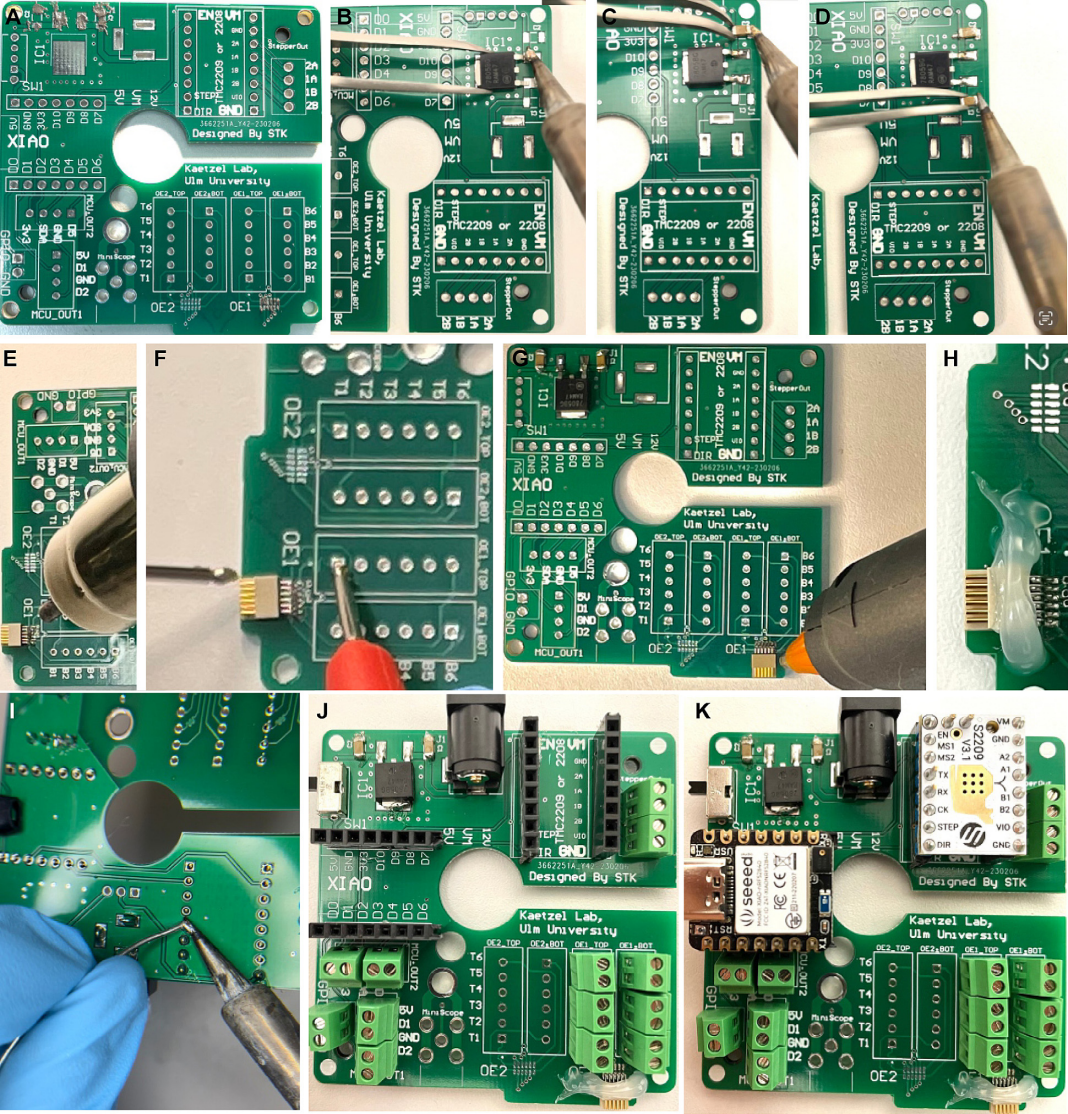
Top PCB preparation. (A) Solder paste applied for SMD components (top-left). (B-D) Soldering linear drop out (LDO) 12-to-5 V regulator (B) and capacitors (C-D) onto PCB. (E) Soldering Omnetics PZN-12-AA connector onto board. (F) Testing of connections between Omnetics connector and through-hole pads using a multimeter. (G-H) Application of hot-gun glue or non-conductive epoxy to Omnetics connector. (G) also shows the whole Top PCB with SMDs fully mounted. (I-J) Soldering through-hole header female sockets, DC jack barrel, and 3-pin header. (K) Mounting stepper motor driver board and XIAO microcontroller.

The top PCB is the main circuit which hosts all controlling units including the XIAO microcontroller with USB-C port, the stepper-motor driver board, and the linear drop-out (LDO) 12 V-to-5 V voltage-regulator, in addition to a DC jack barrel (see Table 3) and the connectors for data recording (Fig. 8A–K). Depending on the application, one can opt for attaching one or two Omnetics^TM^ PZN-12-AA connectors to support either 12 or 24 SPI-channels (ports are labelled OE1 and OE2 on the PCB, see Fig. 8A, G) for electrophysiology recordings and/or one SMA-connector (“miniscope” port in Fig. 8J, SMA connector shown in Fig. 2B,D) for miniscope recordings. Firstly, *Surface Mount Devices* (SMD) are soldered onto the board; this includes the LDO voltage-regulator and capacitors as well as the Omnetics^TM^ PZN-12-AA SPI port(s) (Fig. 2B, 8A–E). For this, either a hot plate or a hot air rework station is used. Note that the LDO and capacitors used here are SMD, instead of through-hole components to benefit from their compact shape and low-cost. Each of the 12 lines of the Omnetics^TM^ PZN-12-AA connector need to be inspected for proper soldering and absence of cross-connection with any of the other lines using a multimeter (Fig. 8F); subsequently the connector is securely fixed to the board using non-conductive epoxy or a hot glue gun (Fig. 2G–H). For commutators to be used for miniscope recordings, the SMA port is soldered onto the board, subsequently (Fig. 2B; not shown in Fig. 8). Then, through-hole female header sockets and 3-pin headers (black in Fig. 8J) as well as output pins are soldered onto the board (Fig. 8I–J). In practice, we have used either terminal blocks for SPI-, Hall-sensor-, stepper-motor- and GPIO-lines (light green in Fig. 8J–K) or direct soldering to through-hole pads for the SPI-lines and JST pins for the other connections (visible in Fig. 12D, F). Among those options, we regard terminal block connectors as the preferrable solution, as they enable a uniform connector standard for the PCB and an easy and reversible connection of the many cables required especially for SPI commutators. Pre-wired JST connectors (e.g. the XH family; part number family ASXHSXH22K from JST Sales America Inc, available through Digikey) would also be a design solution for a uniform standard (not illustrated here), but would be somewhat more time-consuming as they require insulation at the crimping point of every connection to the wires of the slipring.

**Table 3:** Bill of materials. * Distinct numbers are required for these items because their usage depends on the type of commutator that is built. The version of the commutator (specified by small letters in the lower part of the table) is stated behind the number of the required item in the middle part of the table. At the bottom of the table, all 5 types that can be built with the unmodified design files provided here and on https://zenodo.org/record/7640543 are listed and their costs are stated. Also note: Some prices are in USD but have not been converted to Euro given the fluctuation around a 1:1 USD:EUR exchange rate during the preparation of this manuscript. The same table but with the source of all stated materials (as web-link) is provided as Supplementary Table 1.

**Designator**	**Component**	**Number**	**Cost per unit (€)**	**Total cost (€)**	**Manufac-turer Number**	**Material type**
Top PCB	Top PCB	1	1.00	1.00	na	printed circuit board
Bottom PCB	Bottom PCB	1	1.00	1.00	na	printed circuit board
Bottom PCB	Hall Sensor	2	0.30	0.60	na	IC
Top PCB, Bottom PCB	Slide switch	2	0.42	0.84	1825232-1	plastic, metal
Top PCB	Linear voltage drop out regulator (LDO)	1	0.61	0.61	MC7805BDTRKG	IC
Top PCB	Capacitors	2	0.15	0.30	C0603C220K3RACAUTO	ceramic capacitor
Top PCB	Microcontroller (XIAO or XIAO ESP32C3)	1	4.99	4.99	XIAO ESP32C3	IC
Top PCB	DC Power jack barrel	1	1.02	1.02	694106301002	Plastic, Metal
						
Top PCB	Stepper driver	1	10.00	10.00	TMC2208 or TMC2209	IC, PCB
Top PCB	Connector 7P and 8P	4	2.99	11.99	–	plastic, metal
Top PCB, Bottom PCB	Resistors	2	0.44	0.88	MBA02040C1002FC100	metal film resistor
Top PCB, Bottom PCB	2P Terminal Block Connector	25	0.30	7.50	–	plastic, metal
Center case A	Stepper motor	1	22.00	22.00	NEMA14	metal, semiconductor
Magnet holder	Magnet (cylinder 3×4mm)**	2	0.16	0.32	–	Neodynium
Center case A	Ball Bearing (7×42×30)	1	2.60	2.60	6806-2RS	steel
Bottom case B	Ball Bearing (3 × 10 × 4 mm)	1	0.90	0.90	623-2RS	steel
–	M2.5×5.7mm threaded inserts	20	0.12	2.40	–	steel
–	M2.5×25mm Screws	10	0.24	2.40	–	steel
–	M2.5×8mm Screws	20	0.18	3.60	–	steel
–	M3×4mm threaded inserts	4	0.09	0.36	–	brass
–	M3×6mm screws	3	0.07	0.21	–	steel
–	M3×40mm screws	1	0.18	0.18	–	steel
3D printed parts	3D printed parts	1	–	60.00	–	
**Sum total of costs for any commutator type**				**135.82**		
						
**Parts that are ordered in dependence on the specific commutator type to be built**						
Top PCB, Bottom PCB	SMA Connector (Miniscope)*	2 (c, d)	7.13	14.26	73251–2209	metal
Top PCB, Bottom PCB	Omnetics Horizontal SMT PZN-12-AA*	2 (a, d, e) / 4 (b)	42.39	84.78	A79623-001	composite
slip-ring	slip-ring for 12-channel SPI*	1 (a)	20.00	20.00	M220A-18 / M125-18	plastic, conductive material
slip-ring	slip-ring for 24-channel SPI*	1 (b)	75.00	75.00	M220A-36	plastic, conductive material
slip-ring	slip-ring for high-speed RF-line*	1 (c)	85.00	85.00	O022-12 / O022-08	plastic, conductive material
slip-ring	slip-ring for 12-channel SPI & RF-line*	1 (d)	145.00	145.00	O032-24	plastic, conductive material
slip-ring	slip-ring for 12-channel SPI & through-hole*	1 (e)	130.00	130.00	H1532-24S	plastic, conductive material
						
**Sum total of costs for specific commutator type**						
a. Electrophysiology 12-channel SPI (32 recording channels)				**240.60**		
b. Electrophysiology 24-channel SPI (64 recording channels)				**380.38**		
c. Miniscope only				**235.08**		
d. Electrophysiology 12-channel SPI (32 recording channels) and Miniscope				**379.86**		
e. Electrophysiology 12-channel SPI (32 recording channels) and Optogenetics				**350.60**

Then, a slide switch is soldered to the switch port (silver, top-left in Fig. 8J; see also Fig. 2B). In later experimental application this switch can be used to determine the power source that should be used for the stepper-motor; i.e. if the power shall be supplied via the DC-jack (12 V; switch set towards the side of the DC-jack) or the USB-C power source (5 V; switch set towards the USB port). The 12 V DC jack will supply more torque, which is, however, not necessary for proper operation and has not been used regularly by us. Finally, the XIAO microcontroller and stepper-motor driver board (TMC2208 or TMC2209) are mounted onto the female header sockets (Fig. 8K).

### Step 3: Preparing the bottom PCB

**Fig. 9:**
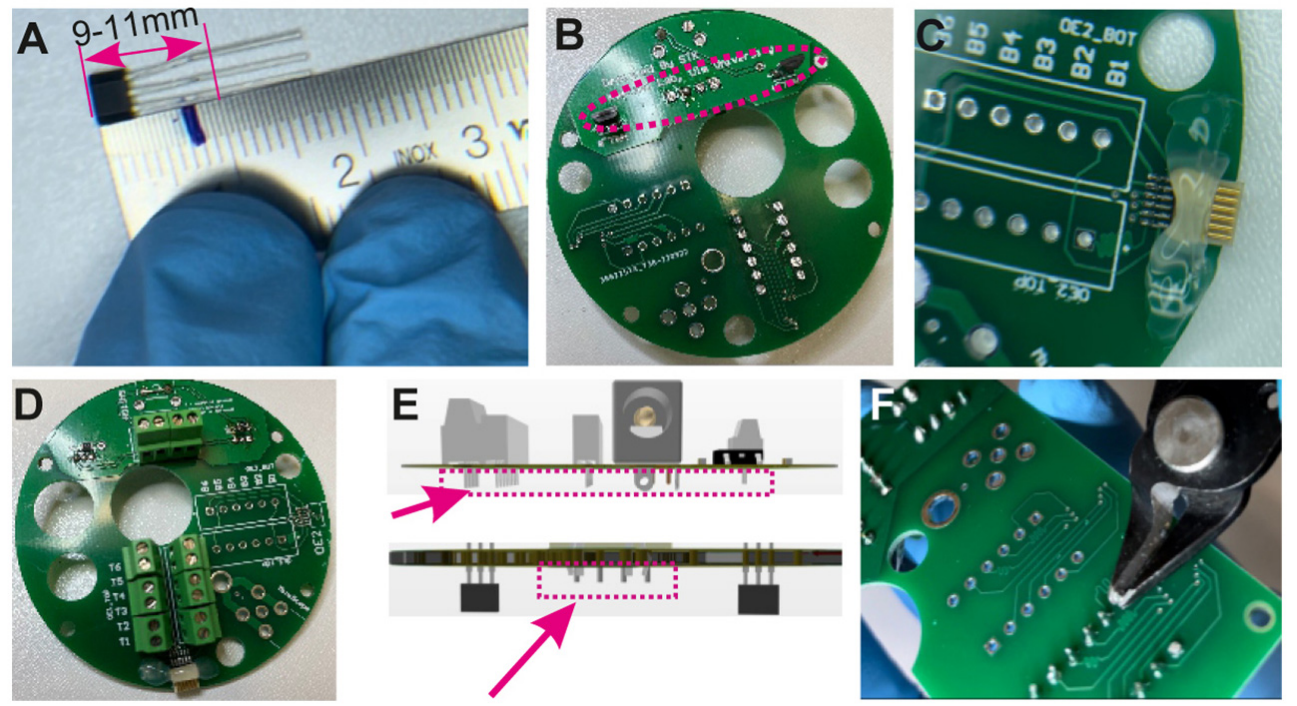
Bottom PCB preparation. (A) Wires of Hall sensor are shortened to required length. (B) Soldering of Hall sensor to the PCB. (C-D) Mounting of Omnetics connector (C) and terminal block connectors (D). (E-F) Trimming of protruding wires at through-hole pins at the lower side of both the top and the bottom PCB.

The bottom PCB hosts the same Omnetics^TM^ and/or SMA connectors as the top PCB in addition to Hall sensors and resistors (Fig. 2C–D). The wires of the Hall sensors are cut to a total sensor length of 9-11 mm and soldered to the respective through-hole pads (Fig. 9A–B). The Omnetics^TM^ PZN-12-AA SPI port(s) and/or SMA port, 6–10 kOhm resistors and terminal block connectors are soldered in the same way as onto the top PCB (Fig. 9C–D). All pins protruding at the bottom of *both* PCBs are cut (Fig. 9E–F).

### Step 4: Preparing the 3D-printed parts for assembly

**Fig. 10:**
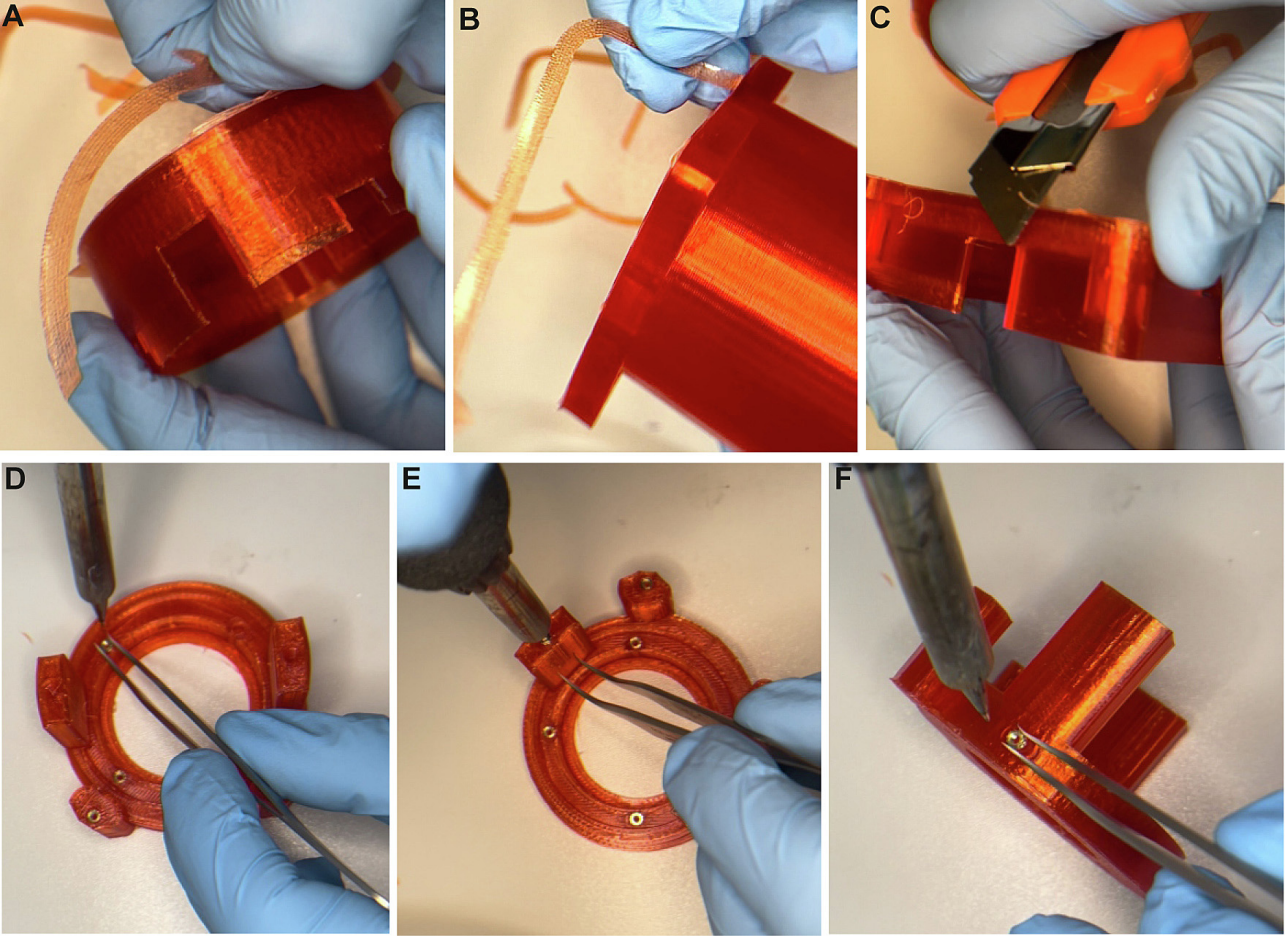
Preparation of plastic case. (A-C) Removal of excess build plate adhesion material. (D-F) Insertion of threaded inserts into mounting holes.

Clear all supporting and build-plate adhesion material from 3D-printed parts (Fig. 10A–C). Then, insert threads into all mounting holes (Fig. 10D–F) using a fine, thoroughly cleaned soldering tip (heated to around 150–200  degreesC for PLA) and forceps. The *slip-ring stage* (see Fig. 4) receives M3×4mm threaded inserts, all other mounting holes require M2.5×5.7mm threaded inserts. See Video 1 for an illustration of this step.

### Step 5: Center assembly

**Fig. 11:**
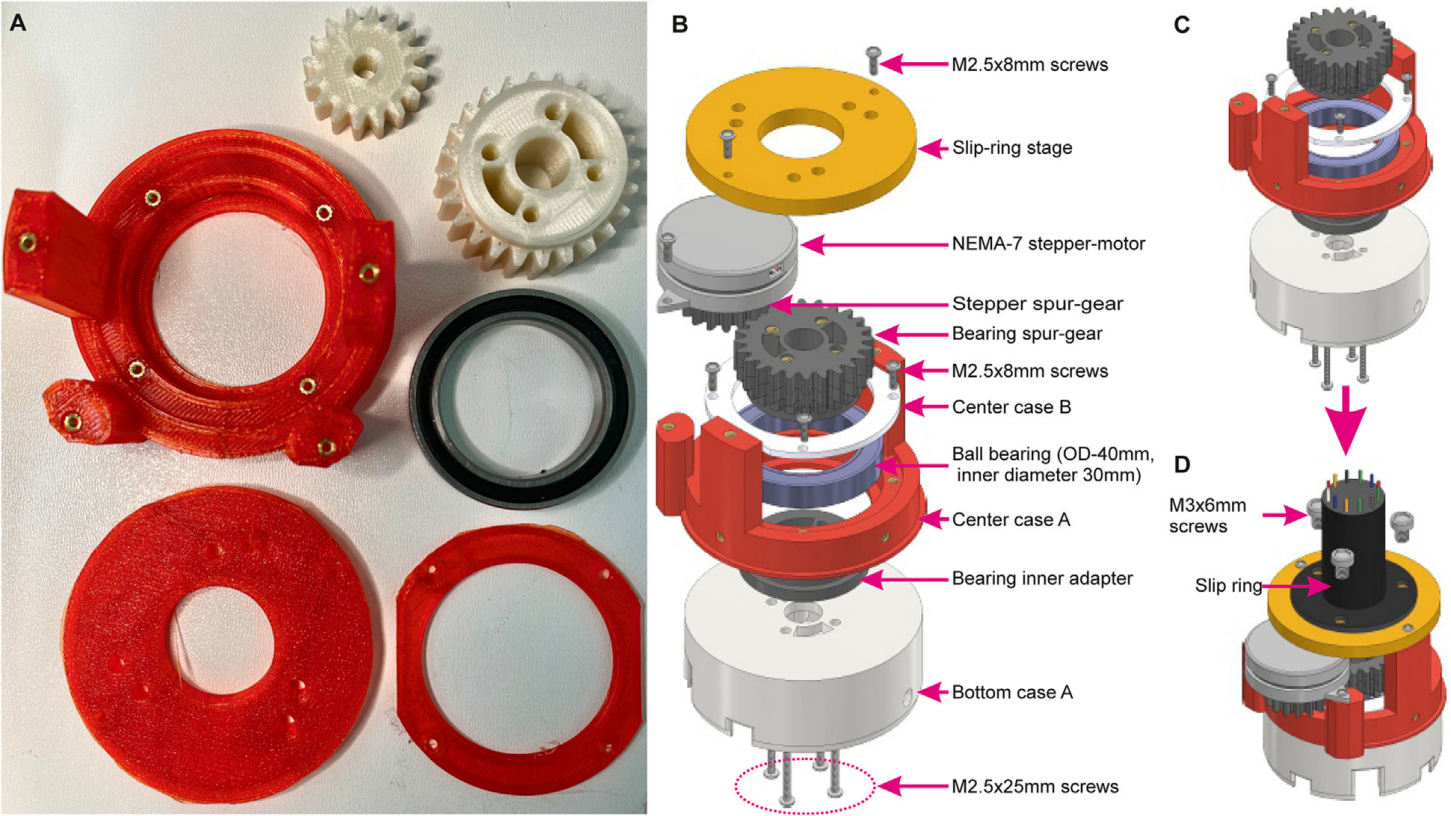
Center assembly. (A) 3D-printed parts with threaded inserts and ball bearing; not including Bottom case A. Also see Fig. 4 for annotation of 3D-printed parts. (B) Overview of center parts showing spatial order of assembly. (C-D) Assembly process for the center of the commutator.

Once all 3D-printed parts are prepared (Fig. 11A), including the threaded inserts (Step 4; Video 1), the ball bearing (7×40×30 mm) is first inserted into the *Center case A* (Fig. 11B). Then, the ball bearing is secured by fixing *Center case B* to *Center case A* using four M2.5×8mm screws (Fig. 11B–C). Subsequently, the bearing spur-gear and the bearing inner adapter are mounted. Then, the *Bottom case A* is fixed to the assmebly using four M2.5×25mm screws as shown in Fig. 11C. Afterwards, the stepper spur-gear is attached to the NEMA14 stepper motor (Fig. 11B) and fixed to the center assembly using two M2.5×8mm screws (Fig. 11B,D). Then the slip-ring is connected to the *slip-ring stage* using three M3×6mm screws (Fig. 11D). See Video 2 for an illustration of this step. Note that the current design of the *slip-ring stage* can fit almost all of the Senring^TM^ models stated in Table 3, except for the model H1532-24S; however, different mounting holes are used for such different slip-ring models depending on the position of their own mounting holes. For model H1532-24S, used for combined optogenetics and electrophysiology the center diameter and hole-positions of the *slip-ring stage* need to be adapted.

### Step 6: Wiring and mounting of top PCB

**Fig. 12:**
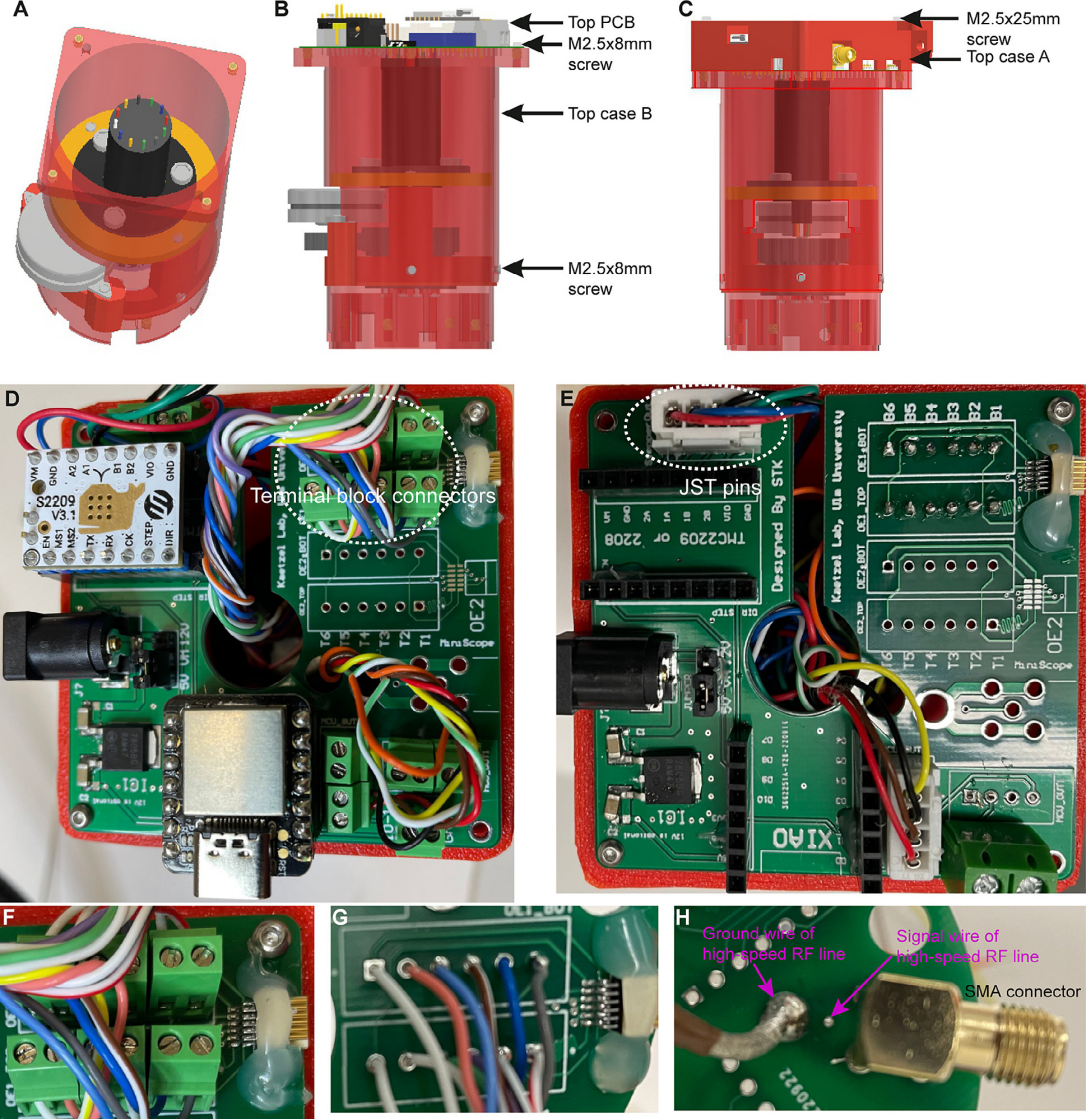
Top case assembly and mounting of top PCB. (A) Overview of top case mounted to center assembly, shown from the top. (B) Top PCB attached to Top case B. (C) Top PCB covered by Top case A. (D-E) Top view onto top PCB mounted to Top case B *with* (D) and *without* (E) stepper-driver and microcontroller boards and terminal block connectors. (F) Enlarged view of SPI-line cables connected to terminal block connectors in OE1-field. (G) Enlarged view of directly soldered SPI-line cables in OE1-field, as an alternative to the arrangement shown in (F). (H) SMA-connector for coaxial cable and high-speed RF line soldered to PCB (needed for miniscope experiments only).

**Fig. 13:**
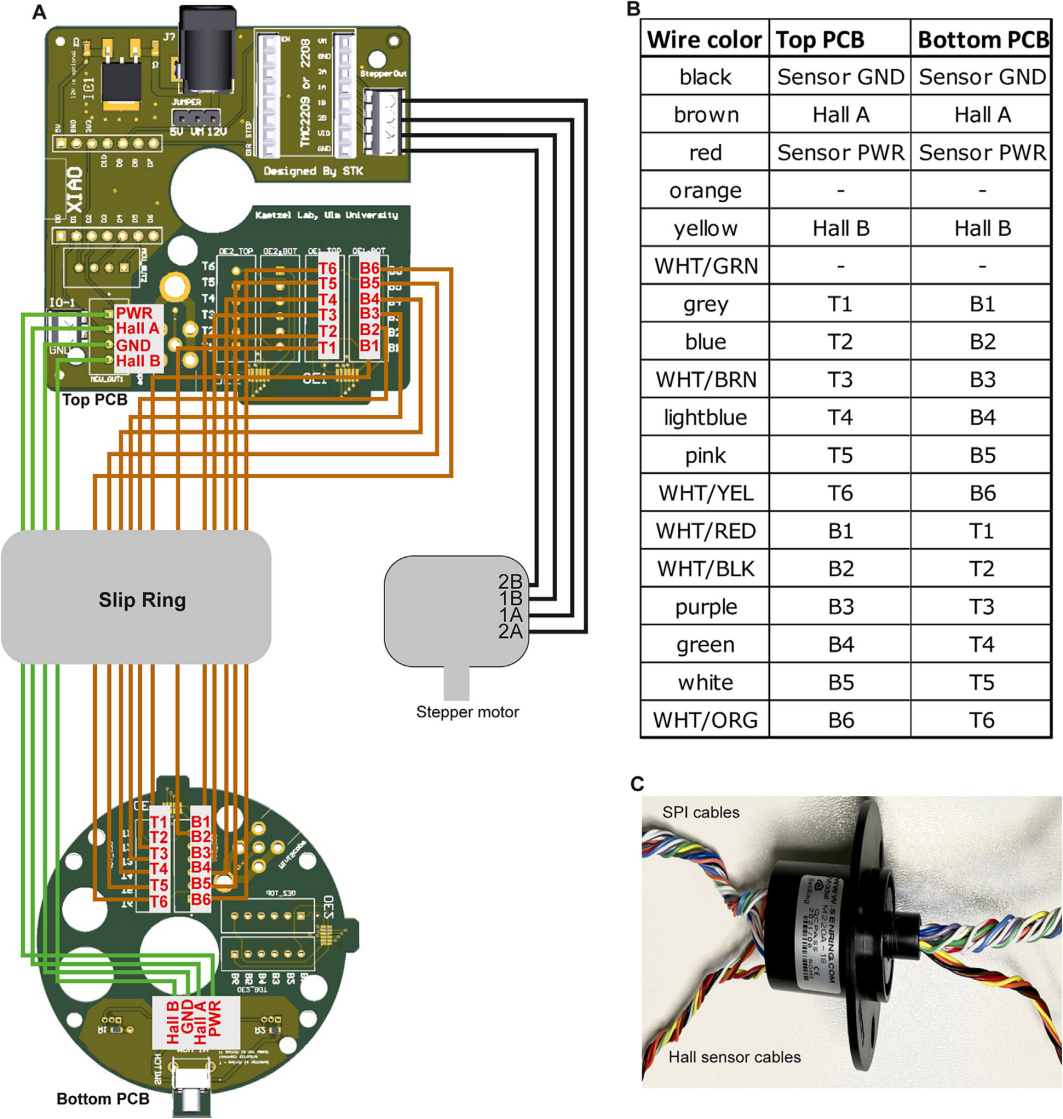
Wiring scheme for electrophysiology. (A-B) Graphical (A) and tabular (B) display of the connection scheme for SPI, Hall-sensor and stepper-motor lines, as required for electrophysiological recordings with up to 32 channels. (C) Prepared slip-ring (shown here is the Senring M220A-18) with the 12 SPI-lines and the 4 Hall sensor lines twisted as separate groups.

**Fig. 14:**
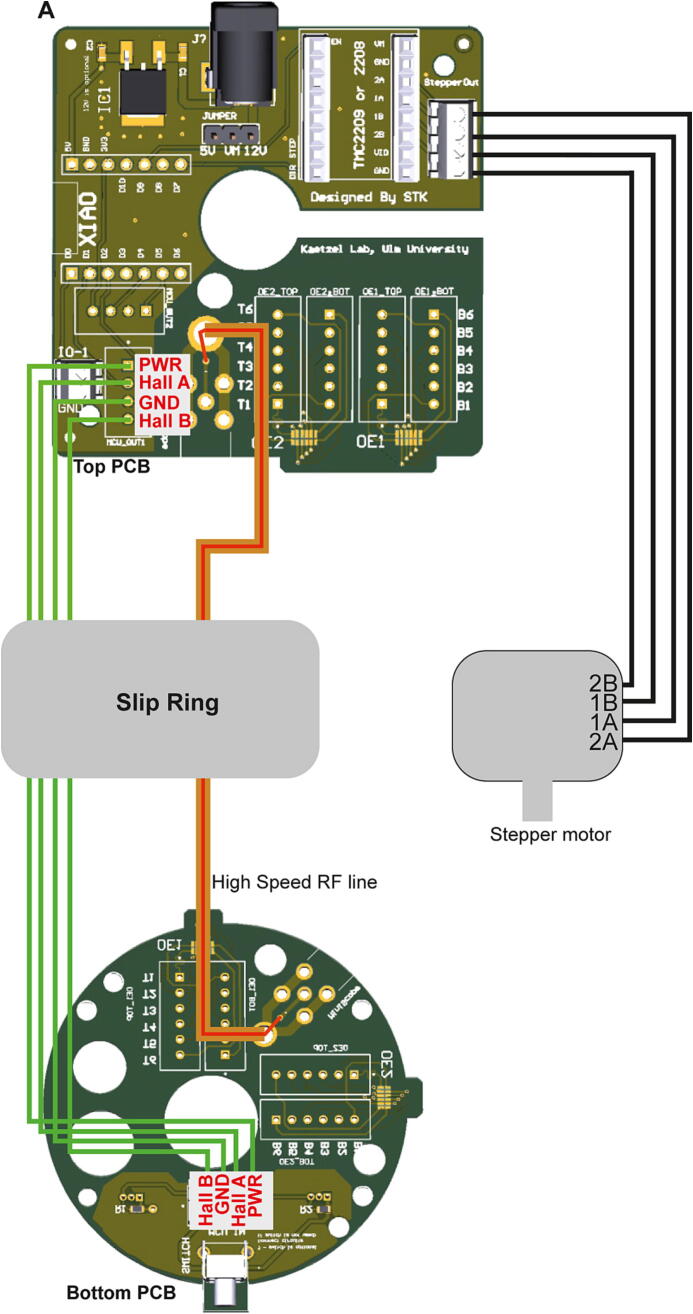
Wiring scheme for miniscope. (A) Graphical display of the connection scheme for high-speed RF, Hall-sensor and stepper-motor lines, as required for miniscope recordings using a single-channel coaxial cable.

The *Top case B* is connected to the center assembly using three M2.5×8mm screws and the top PCB is put on top of *Top case B* (Fig. 12A–B). Then the wires that represent the SPI lines are inserted into the terminal block connectors, if present (Fig. 12D, F), or are soldered directly to the respective through-hole pads of the OE1 (and, if 24 SPI-lines are used, the OE2) field (Fig. 12E, G) according to the scheme shown in Fig. 13A–B. If the commutator shall be used for miniscope recordings, the SMA-connector should have been attached to the “miniscope” field before (Step 2); then the high-speed RF-cable from the slip-ring needs to be connected to this field by soldering the exposed ground wire end to its ground pad and the center signal wire to the smaller signal pad in front of it (Fig. 12H); if the commutator is used only for miniscope recordings, no SPI-lines and –connectors are involved and the wiring scheme of the PCB is therefore reduced as shown in Fig. 14. Next, the stepper-motor wires are connected to the stepper-motor driver output ports as shown in Fig. 12D (via terminal block connector) or E (via JST pins) and according to the wiring scheme shown in Fig. 13, Fig. 14. Equally, through-hole pads for the Hall sensors including their power and ground cables are connected to the corresponding wires from the slip-ring either via terminal block connectors or JST pins (Fig. 12D–E) according to the wiring scheme shown in Fig. 13, Fig. 14. Note: if the stepper-motor cables and Hall sensor cables are connected using JST pins instead of terminal block connectors, special crimping pliers to make JST pins are required. Finally, the fully wired top PCB is fixed to *Top case B* using two M2.5×8mm screws at diagnonal positions (Fig. 12B, top), and *Top case A* is mounted on top of the PCB using two M2.5×25mm screws fixed to the remaining two mounting holes on the *Top case B* (Fig. 12C). See Video 3 for an illustration of this step.

### Step 7: Wiring and mounting of bottom PCB

**Fig. 15:**
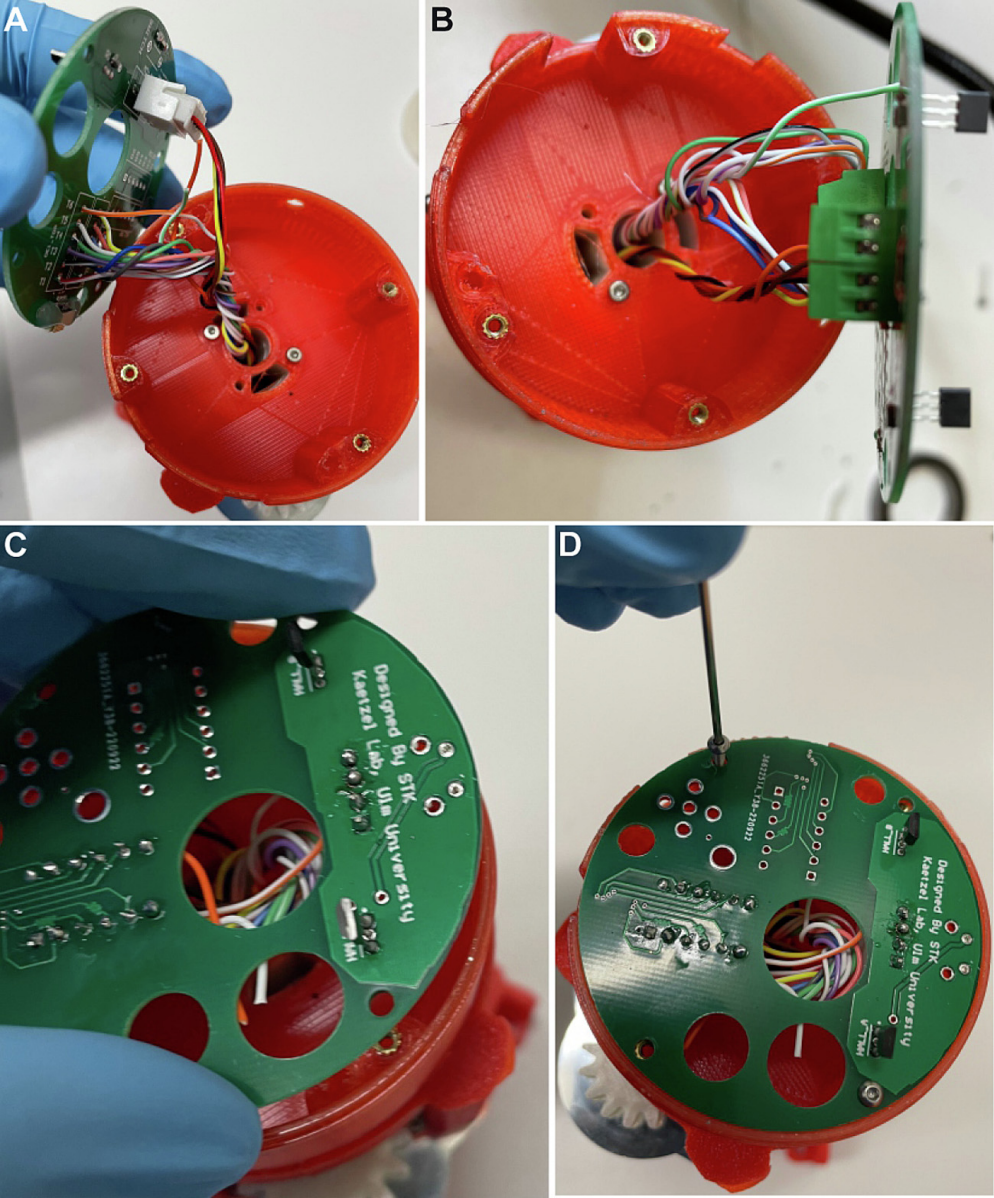
Connection of bottom PCB to center assembly. (A-B) Bottom PCB connected to 12 SPI-cables and 4 Hall-sensor cables (protruding from Bottom case A of center assembly), either using soldering into through-hole pads for SPI-lines and JST-pins for sensor cables (A) or using terminal block connectors (B). (C-D) Fixation of bottom PCB to Bottom case A of center assembly using M2.5×8mm screws.

Analogously to the wiring of the top PCB, SPI-lines from the slip-ring of the center assembly (pro-truding from the lower end of *Bottom case A*, Fig. 11B) are connected to the bottom PCB either via soldering to the corresponding through-hole pads (Fig. 15A) or via terminal block connectors (Fig. 15B); analogously, the Hall sensor cables from the slip-ring are connected via JST pins (Fig. 15A) or terminal block connectors (Fig. 15B). For commutators used for miniscope experiments, the high-speed RF-line protruding from the slip-ring needs to be connected to the miniscope field, and thereby to the SMA-connector of the bottom PCB (not shown). As for the top-PCB, the connection scheme shown in Fig. 13, Fig. 14 should be followed for this process. Subsequently, the wired bottom PCB is secured to the *Bottom case A* of the commutator assembly using two M2.5×8mm screws at diagonal positions (Fig. 15C–D; Video 4).

### Step 8: Software installation and uploading to commutator microcontroller

*Arduino Integrated Development Environment* (IDE) is downloaded from the Arduino^TM^ website (https://docs.arduino.cc/software/ide-v1/tutorials/Windows) and installed onto a host PC used for programming the commutator microcontroller (this can be the same as the acquisition and recording computer used later for experiments). Additionally, *Seeeduino Stalker V3* is downloaded from https://wiki.seeedstudio.com/Seeed_Arduino_Boards/ and added to IDE as per the instructions provided by this website. Furthermore, the specifications file “sketch_torque_commutator.ino” which is provided as Supplementary File 1 (pdf) and 2 (actual.ino file to use) and on GitHub (https://github.com/KaetzelLab/Open-MAC) should be downloaded to the host PC.

**Fig. 16:**
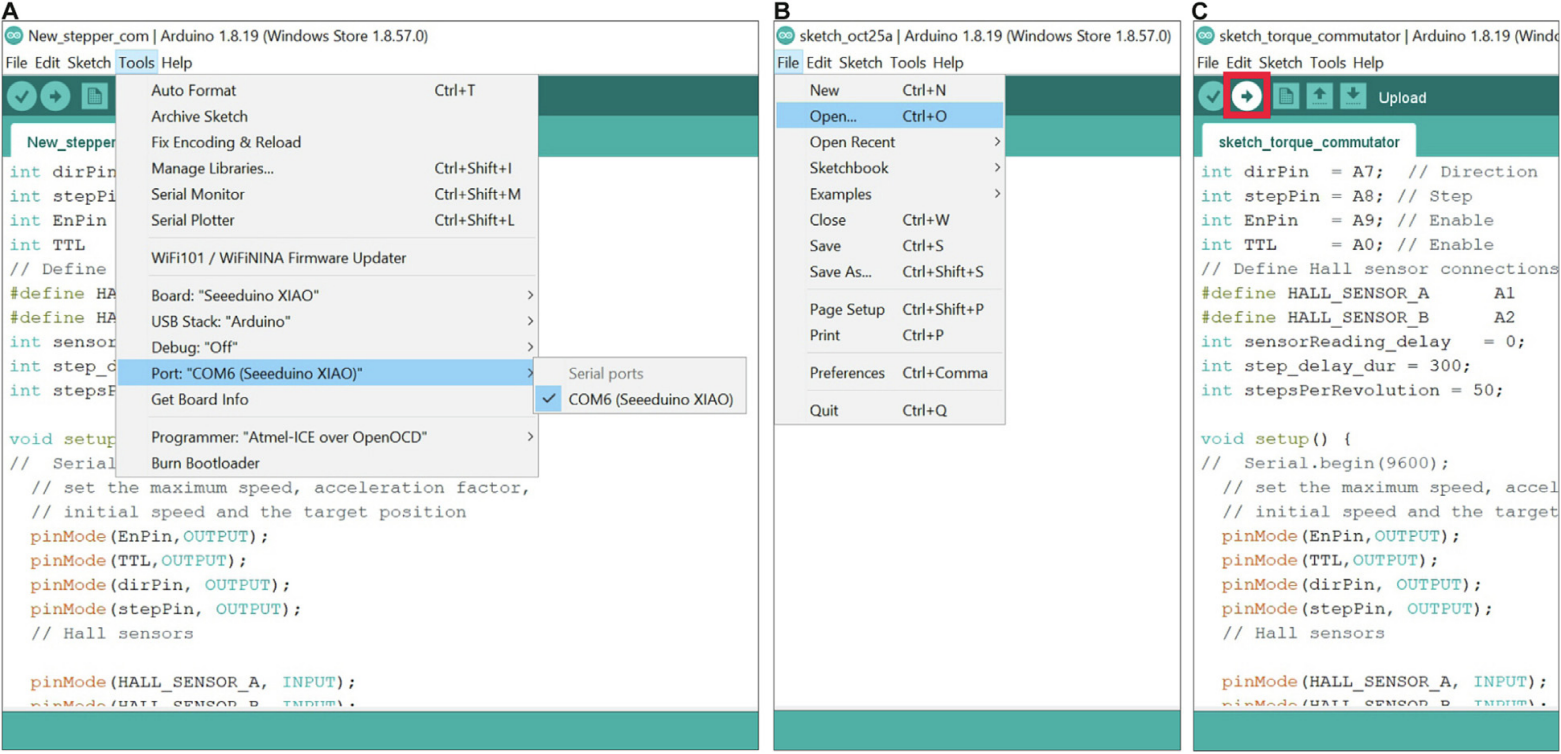
Installation and usage of Arduino IDE. (A) Selection of commutator microcontroller as target COM-port for code upload in Arduino IDE. (B) Opening of.ino code-file from the source folder of host PC. (C) Uploading of code to commutator microcontroller by pressing the arrow-button marked in red. (For interpretation of the references to colour in this figure legend, the reader is referred to the web version of this article.)

The USB-C port of the commutator (microcontroller) is connected to a normal USB2- or USB3-port on the host PC. Then Arduino IDE is opened and the “Tools” main menu is chosen to find and select the “Port” corresponding to the Seeeduino XIAO in the drop-down menu (Fig. 16A); the board will have a COM target serial number (COM) which will be addressed in the following step. Subsequently, the “File” main menu of Arduino IDE is selected to press “Open…” and choose the “sketch_torque_commutator.ino” file (Supplementary File 2) previously copied onto the host PC. Finally, this file is uploaded to the XIAO microcontroller by clicking the “Upload” (rightward arrow) icon in the main menu (Fig. 16B).

### Step 9: Identification of magnet orientation and inspection of wire-connections

Once the upload is done, the individual magnet is held close to each of the two Hall sensors; this should cause the commutator to turn left when the magnet is close to the left sensor (and to the right when the magnet is close to the right sensor). The magnet poles which cause this movement when facing towards the Hall sensor are marked as they also need to face the sensor in the final assembly. This procedure also confirms the functionality of the Hall sensors.

Additionally, each of the 12 SPI-cable connections – that are now running from the top PCB via the slip-ring to the bottom PCB – is checked for a proper connection between the corresponding ports (according to the scheme shown in Fig. 13) and for unwanted cross-connections to the other, non-matching ports using a multimeter (e.g. the bottom PCB’s bottom row should be connected to the top PCB’s top row; Fig. 13A). If there is a cross-connection or a missing connection, steps 6 and 7 need to be repeated.

### Step 10: Preparation of the bottom assembly and magnet mounting

**Fig. 17:**
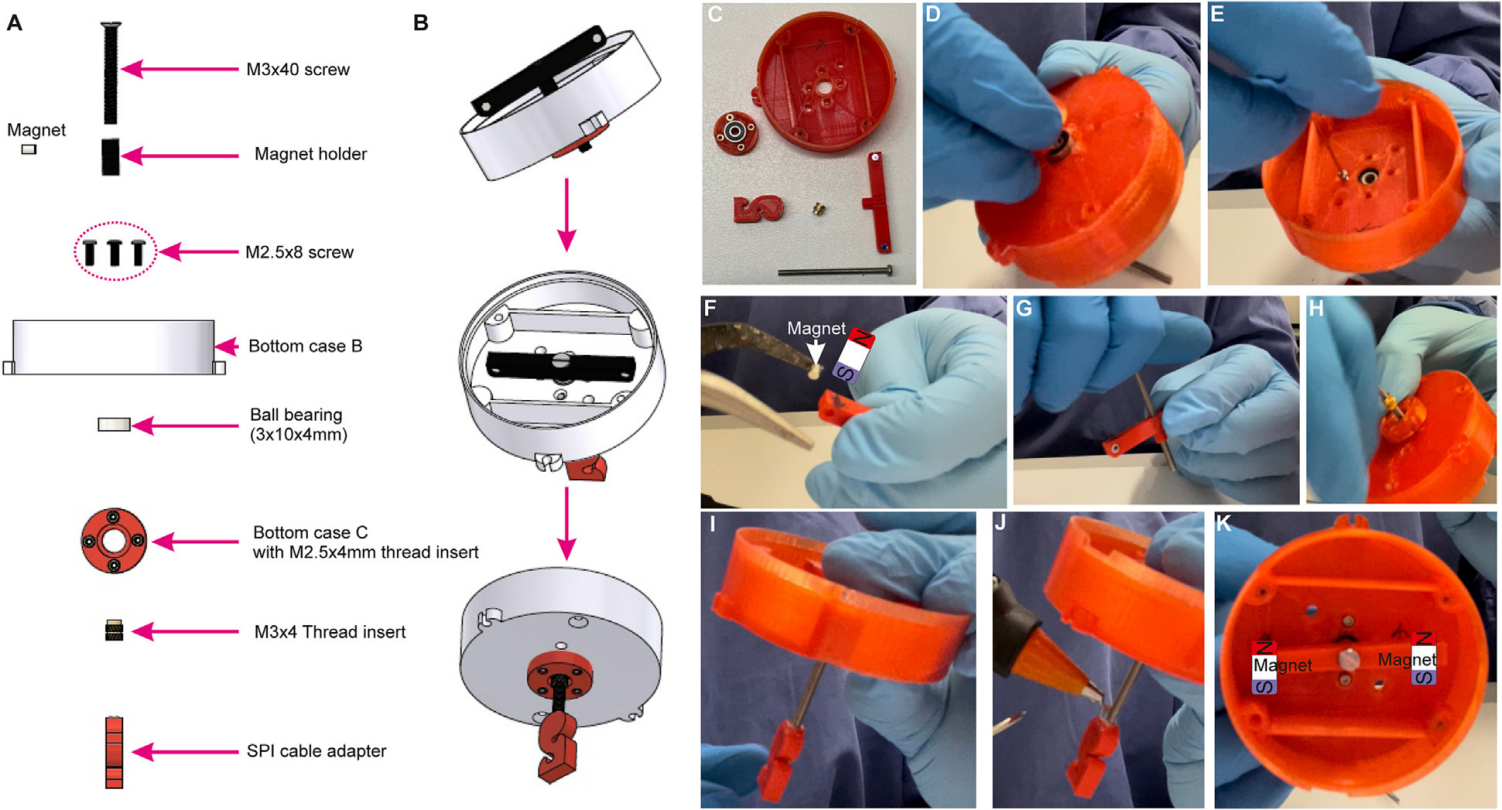
Bottom assembly. (A) Overview of components of commutator bottom, excluding bottom PCB, in spatial order of assembly. (B) Schematic of stepwise assembly process from top to bottom; lower two panels show the same assembly stage from two different perspectives. (C) 3D-printed parts, threads and screw for bottom assembly. (D-J) Individual steps of bottom assembly; the orientation of the magnet is crucial for later operation. (K) Fully assembled bottom part viewed from top with magnets positioned properly in Bottom case B.

**Fig. 18:**
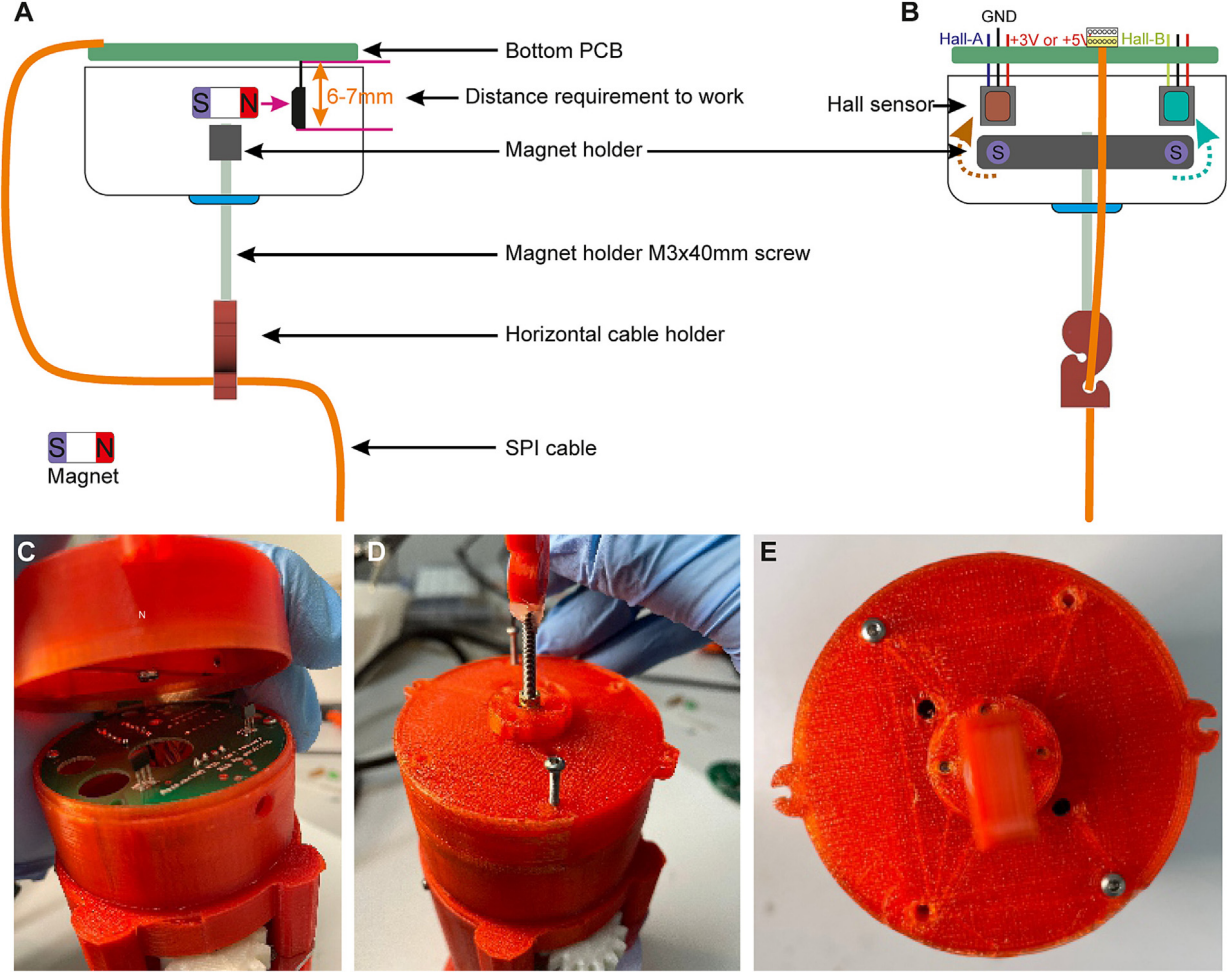
Fixation of properly oriented bottom assembly to center assembly. (A-B) Schematics of bottom assembly illustrating position of Hall sensor, magnet holder, and magnets as well as of horizontal cable holder from two different side views (A and B), rotated by 90 degrees relative to each other. (C) Combining bottom assembly (top) and center assembly with magnets being properly oriented relative to Hall sensors. (D-E) Fixation of both assemblies with M2.5×25mm screws to yield fully assembled commutator (as shown in Fig. 1B).

The *Bottom case B*, the ball bearing and the *Bottom case C* are assembled together using four (or two diagonally opposing) M2.5×8mm screws (Fig. 17A–E). Then, the magnets are placed into the *Magnet holder’s* mounting holes using cutting pliers (Fig. 9F); thereby the two magnets need to be oriented in same polarity direction (see Fig. 17K). Subsequently, the M3×40mm screw is inserted into the center of the *Magnet holder* (Fig. 17G) and this assembly is screwed to the lower end of the bottom assembly using the same screw and M3×4mm threaded inserts (Fig. 17H). Next, the *Horizontal cable holder* is fixed to the bottom end of the same M3×40mm screw (Fig. 17I), whereby the cable holder’s orientation should be as shown in the Fig. 18A–B, before it is fixed with glue (Fig. 17J). The top view onto the final bottom assembly is shown in Fig. 17K. See also Video 5.

### Step 10: Final assembly

With the *Magnet holder* being oriented in parallel to the two Hall sensors and the magnets’ North pole facing those sensors (Fig. 18A–B), the top part of the bottom assembly should be placed onto the bottom part of the center assembly in an inverted way as shown in Fig. 18C. The two parts are connected to each other using two diagonally opposing M2.5×25mm screws (Fig. 18D–E; Video 6).

## Operating instructions

### Torque based operation

Once assembly is done, operation is straight forward:

1. The commutator is fixed in vertical position above the center of the behavioural arena to be used for experiments (Fig. 19).

**Fig. 19:**
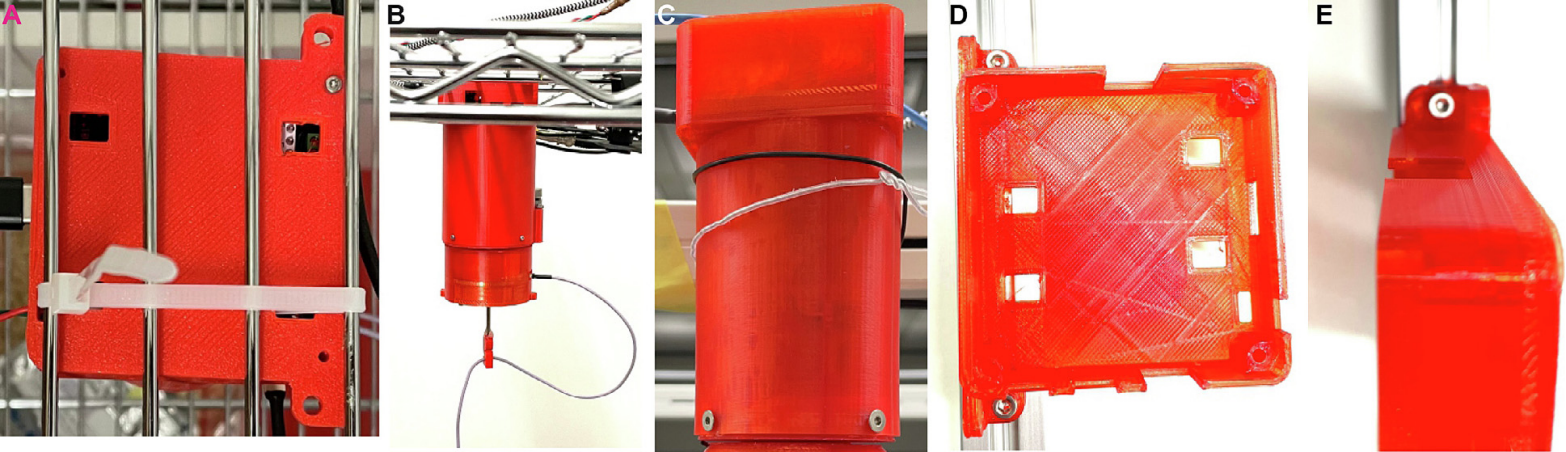
Options for attachment of the commutator. (A-B) Top view (A) and side view (B) of the commutator mounted onto grid of a metal shelf that also hosts the behavioural apparatus below (not shown) using a cable tie passing through top case holes. (C) Attachment using wires that go around the slim center body part and connect to a horizontal rod at the back of the commutator. (D-E) Mounting with M3 screws passing through side holes of the top case and fixing the commutator onto a horizontally protruding 20×20 mm aluminum rail.
2. For electrophysiology experiments, the top PCB’s Omnetics^TM^ PZN-12-AA connector is connected via an SPI-cable (using the same connectors) to an Open-Ephys or Intan^TM^ acquisition board (https://open-ephys.github.io/acq-board-docs/, https://intantech.com/RHD_system.html). For miniscope experiments, its SMA-port is connected to the miniscope DAQ box [7] via a coaxial cable.
3. Similarly, the bottom PCB’s PZN-12-AA or SMA-port is connected to an SPI- or coaxial cable, respectively, which connects to the electrophysiology headstage or miniscope, respectively. In either case, the cable should be put into the corresponding hole of the *Horizontal cable holder* (Fig. 1B) with the length of the protruding cable being adjusted so that the animal can just reach the most remote location of the arena. If the lower cable is too long, it can be wound up horizontally around the *Bottom case B* before passing through the *Horizontal cable holder*. Note that the cable part between the lower SMA/Omnetics^TM^ connector to the *Horizontal cable holder* should be at least 20 cm to form a loop (Fig. 1B; Video 7–8).
4. The commutator is then connected either via its USB-C port to the acquisition laptop or via the DC jack to a separate 12 V DC adapter to provide the power for its operation. The commutator will instantly operate depending on the torque exerted onto the lower cable by the animal. Once the animal starts to rotate, the cable’s torque will move the *Magnet holder* left or right which will activate the Hall sensor to turn the bottom part of the commutator into the same direction to counteract the torque.

### Torque-free operation

The connection and activation of the commutator is done in the same way as described above for the *torque-based mode* (steps 1–4), although a further cable may have to be used for transmission of positional information (see below). The torque-free mode is an advanced, yet to be fully developed and tested option which allows the user to control the commutator’s rotation by sending digital or analogue signals to the commutator’s microcontroller via Bluetooth, USB-C, or GPIO. The rotation information can be extracted either from deep-learning based online pose estimation software such as the *DLC live* [5] or *DeepLabStream* [6] or from accelerometer data using *Bonsai*. For more information on the implementation of pose estimation and processor design, refer to the instructions for creating the respective processor [8], [9] which needs to be modified for application with the commutator. Detailed direct implementation of this advanced system is still under development by our laboratory and will be provided on https://github.com/KaetzelLab/Open-MAC.

## Validation and characterization

We have so far used the presented commutator – or various prototypes – in the *torque-based* mode for several hundred hours of electrophysiological (with 12-channel ultra-thin SPI) or miniscope recordings in awake mice in different behavioural arenas using cable-length between approx. 70–130 cm between the mouse and the *Horizontal cable holder*. In these applications, the Open-MAC reliably reacted to rotations of the animal even with minimal torque (Video 7–8) allowing virtually walk-away-safe experimentation with no manual interference for untwisting. We have not applied or tested the Open-MAC in the *torque-free* mode, as this is a much more specialised application which might be of use for some labs but requires considerable further developments on the corresponding software side to extract information about the animal’s rotations; we envision that the easy, stand-alone usability of the commutator in *torque-based* mode will be the most useful for the vast majority of researchers and projects.

**Fig. 20:**
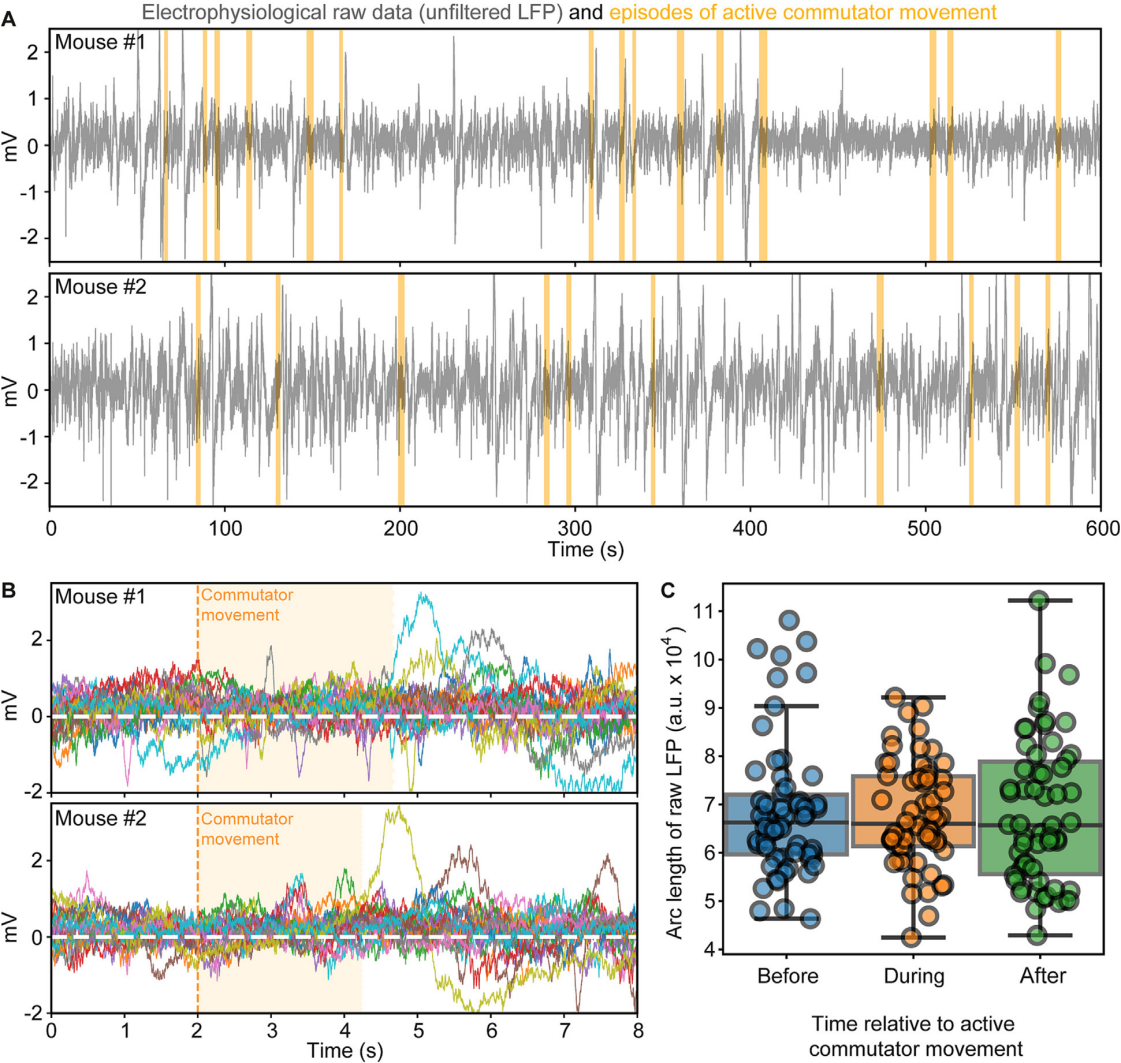
Validation of electrophysiological recordings with Open-MAC based on signal amplitudes. (A) Example unfiltered, raw local field potential (LFP) traces recorded in prefrontal cortex of two individual mice (top and bottom) over 10 min, down-sampled to 1 kHz, with corresponding simultaneous recording of commutator rotations highlighted in orange (derived from TTL-input). Note that rotations did not cause interruptions or artifacts of the recording. Surgeries and recordings were done as previously described [10]. (B) Data from the same two mice, but unfiltered traces from 20 randomly selected episodes of active rotations of the commutator (in different colours) are shown time-locked to the onset of the activation (dashed vertical line). The median duration of the activation of the commutator is illustrated by the light-orange background. Episodes have been selected from the total recording time of 20 min per mouse. (C) Coastline (arc length) values of every episode of commutator activation recorded in the same two mice calculated separately in the 2 s before, during (starting from the onset), and after the rotation event. No statistical difference was found between the three time points (P > 0.5; paired t-test). (For interpretation of the references to colour in this figure legend, the reader is referred to the web version of this article.)

**Fig. 21:**
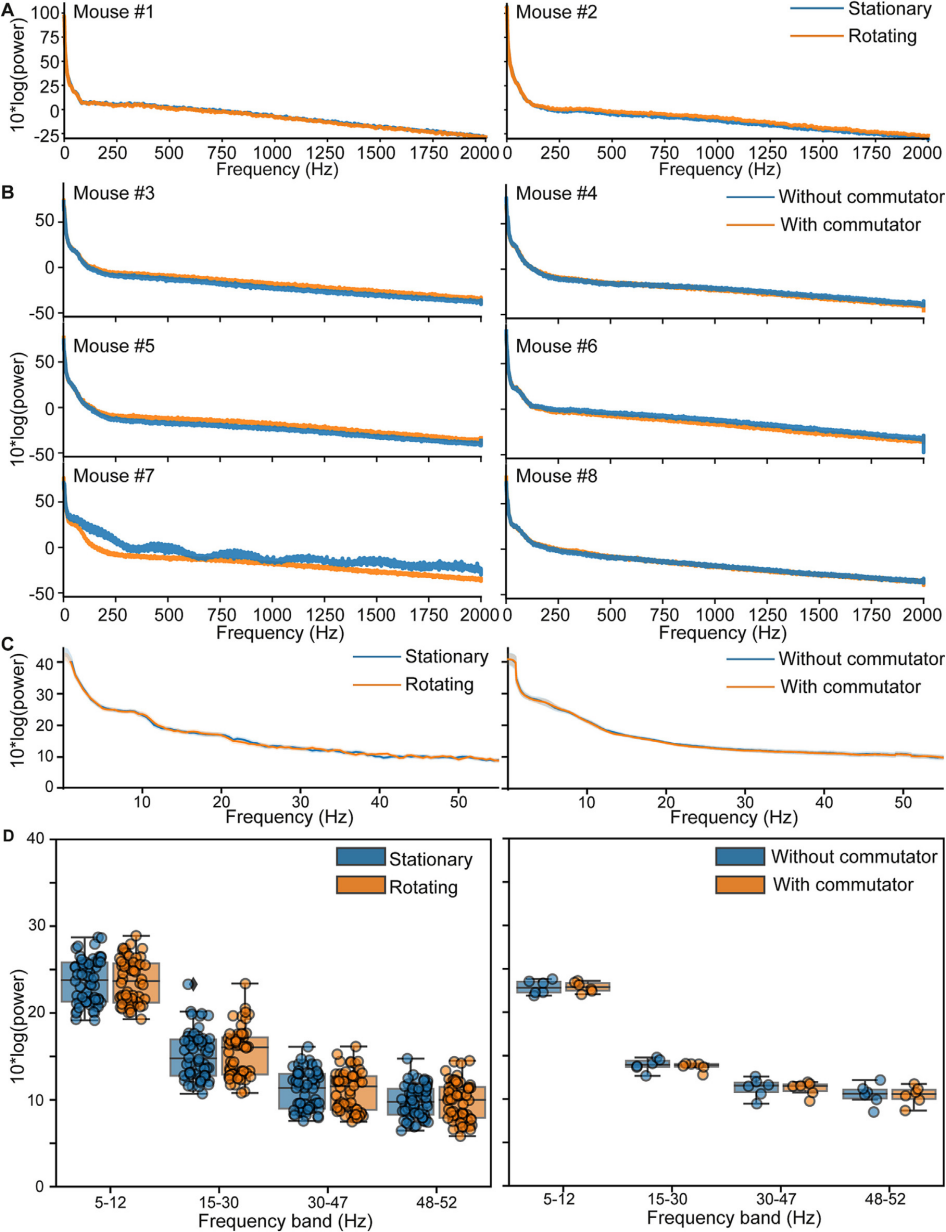
Validation of electrophysiological recordings with Open-MAC based on power spectra. (A) Average power spectra calculated between 0.1 and 2000 Hz, as previously described [10], for 2 s episodes when the commutator was actively rotating (stepper motor active) or stationary immediately before rotation onset (data shown for individual mice). (B) Average power spectra calculated between 0.1 and 2000 Hz for recordings made with or without the commutator in the acquisition pipeline in the same six mice, shown individually. Data was down-sampled to 5 kHz before calculating power spectra. (C) Average power spectra calculated between 0.1 and 52 Hz, either for episodes when the commutator was actively rotating (stepper motor active) or stationary (left; data from mice #1–2), or with or without the commutator in the acquisition pipeline (right; data from mice #3–8), respectively. Shaded regions display s.e.m. (D) Same data as in (C) but displayed for all individual episodes of the same condition individually (dots) and statistically (box-plots), and averaged within relevant frequency bands (x-axis). No statistical differences between the shown commutator-related conditions were found in the dataset (paired t-test).

The major concern with incorporation of a commutator in the data acquisition line is interruption of recording and introduction of noise. We have therefore analysed two of our recorded electrophysiology datasets regarding these concerns; six adult female mice had been operated and recorded as described previously [10]. In one dataset, we simply used the GPIO-line to send TTL-pulses whenever the commutator’s motor was activated. We analysed 20 min of recordings from a total of two mice in an open-field, which included 60 episodes where the commutator was actively rotating. Visual inspection of 10 min of raw data recorded from the prefrontal cortex (PrL) of each mouse confirmed the absence of artifacts during the activation of the motor (Fig. 20A). To further confirm this, we plotted the unfiltered raw data time-locked to the onset of each rotation event for each mouse (Fig. 2B) and calculated the line integral (also known as coastline or arc length) for 2 s before, during and after each rotation event (Fig. 2C), noticing no differences between active and quiet episodes of the commutator. Using data from the same electrodes, we calculated power-spectra (as described previously [10]) within the frequency range of 0.1–2000 Hz averaged across episodes where the commutator was either stationary or rotating; spectra were virtually identical, irrespective of the commutator’s activity (Fig. 21A).

Secondly, we analysed a dataset, from recordings from the PrL region of 6 awake behaving mice in an open-field which were conducted once *with* and once *without* the functioning and powered commutator in the acquisition line (5 min per mouse; order of condition counterbalanced across mice) to test, if the commutator itself adds any noise (irrespective of its operation). We conducted the same power-spectral analysis as for the first dataset, albeit in this case calculating individual power values for the whole recording in one experiment. Again, we found no difference (except that one power spectrum from a recording done *without* commutator appeared more noise which likely relates to the visually observed strong twisting of the cable, Mouse #7; Fig. 21B). To confirm this result statistically, we averaged the power spectra across animals for each dataset (Figure C) and compared the average power in the biologically relevant frequency bands (theta, 5–12 Hz; beta, 15–30 Hz, low-gamma, 30–47 Hz) and for 50 Hz grid noise (48–52 Hz) across the population of stationary and rotating episodes or recordings with and without commutator, respectively, but found no difference (*P* > 0.5; *t*-test; Fig. 21D).

**Fig. 22:**
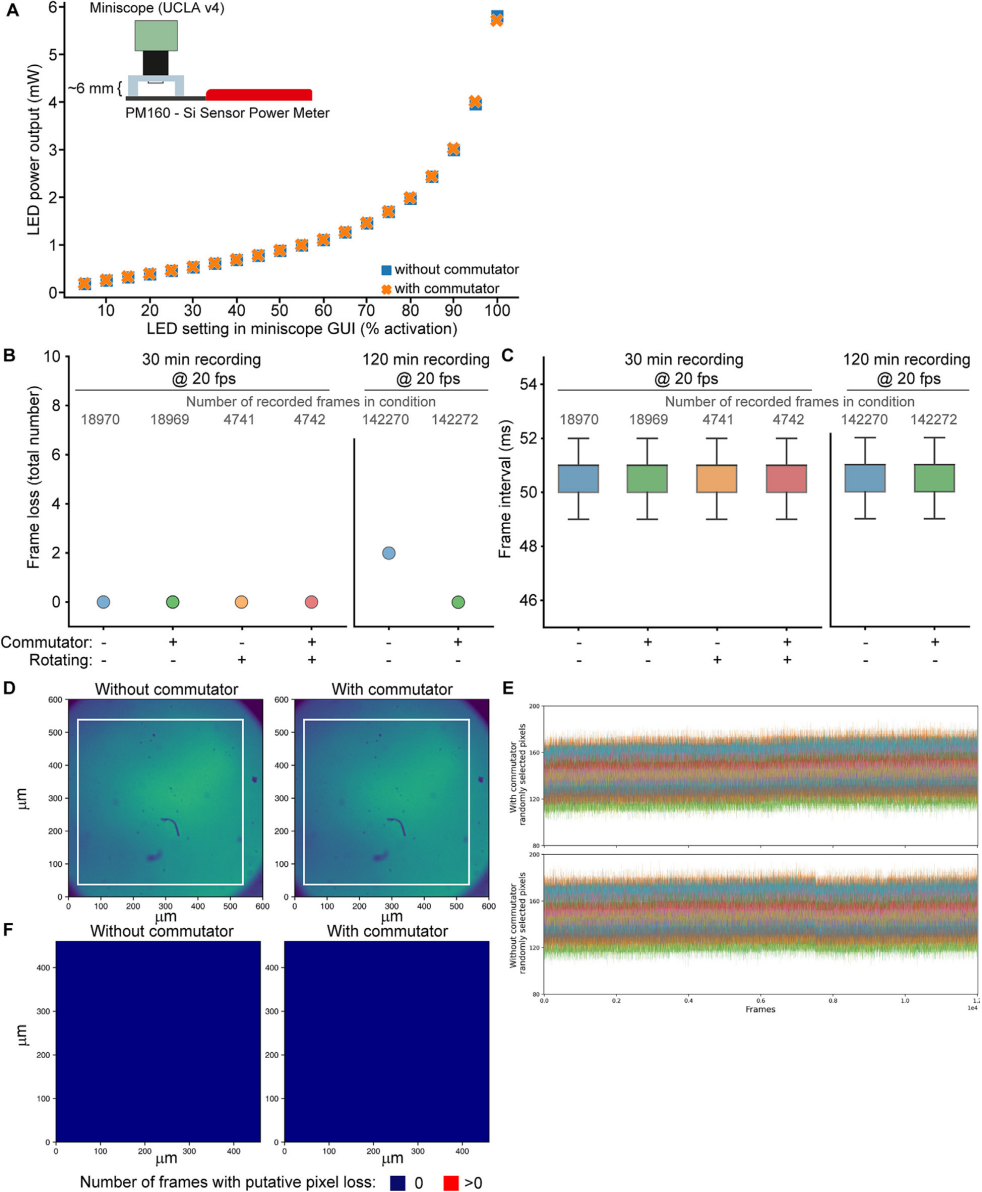
Validation of miniscope recordings with Open-MAC. (A) Optical output power of excitation LED of a UCLA v4 miniscope recorded over the full scale of available output settings with a power-meter at approx. 6 mm distance once *with* and once *without* the commutator in the acquisition line. (B) Number of lost frames out of the total number of recorded frames stated in grey during recordings made for 30 min (left) or 120 min (as indicated at the top) at 20 fps *with* or *without* the commutator in the acquisition line, and *with* or *without* rotation of the sample and the attached UCLA v4 miniscope (indicated on the x-axis). Rotations occurred every 30 s for 4 s in alternating direction; for the *rotating* conditions, only frames recorded during rotations were analysed (60 rotations, 240 s), leading to a lower frame count. Note that only 2 frame losses were detected across all experiments, and these occurred during a recording *without* commutator. (C) Data from the same experiment as in (B), but here the distribution of frame intervals is shown as box plots (box indicates 25–75% interquartile range, IR, and whiskers extend for ± 1.5 × IR). The inter-frame interval was slightly longer than the 50.0 ms expected at 20 fps in all conditions (average = 50.607 ms), leading to somewhat lower frame numbers than expected in panel (B). (D) Average image across 12,000 frames obtained from a recording of approx. 10 min duration of a sample of 1:10-diluted green-fluorescent RetroBeads^TM^ (LumaFluor Inc, US) once *with* and once *without* the commutator in the acquisition line. The sample and the attached UCLA v4 miniscope was rotated intermittently; rotations occurred every 6 s for 4 s in alternating direction. The white square indicates the field of view (FOV) used for further analysis depicted in (E-F) to avoid dark borders of the sample, and includes 202,500 pixels. (E) Amplitude levels at 50 randomly selected pixels in the FOV shown in (D) (encoded by distinct colours) over 10 min recording time with intermittent rotation conducted either *with* (top) or *without* (bottom) commutator. Pixel failures would be detectable by drop of individual lines towards an amplitude of 0, which is not seen. (F) Same recording as in (D); for the inner FOV (indicated by the white square in (D)) the number of frames where the amplitude value of a given pixel dropped below 70% of the over-time average signal amplitude of that pixel (indicating putative pixel loss) is encoded by colour. None of the pixels failed in any frame. (For interpretation of the references to colour in this figure legend, the reader is referred to the web version of this article.)

To validate the commutator version with the high-speed RF-line for usage with the UCLA miniscope, we conducted several technical tests, always using the same v4 miniscope. Firstly, we confirmed that the inclusion of the commutator in the data acquisition line does not change or limit the power transmission that is critical to illuminate the LED. Using a light power meter (PM 160, Thorlabs, DE) positioned at a fixed distance from the miniscope (approx. 6 mm) we recorded the LED output power relative to the setting in the miniscope GUI once *with* and once *without* the commutator. The resulting output power was virtually identical (Fig. 22A). Next, we recorded imaging data at 20 fps over either 30 or 120 min, in different conditions, namely *with* or *without* commutator, and *with* or *without* rotation of the specimen to which the miniscope was stably attached to. We detected no frame loss in any condition involving the commutator, and the inter-frame interval was virtually identical between conditions, indicating that the commutator does neither corrupt nor delay frame acquisition (Fig. 22B–C). Finally, we recorded a specimen of diluted green-fluorescent beads (Green RetroBeads™ IX; LumaFluor Inc, US) stably attached to the miniscope over a period of approx. 10 min with intermittent rotations lasting 4 s and occurring every 6 s (Fig. 22D). Signal amplitudes of randomly selected pixels did not show a noticeable drop towards 0 at any time point, which would indicate a pixel failure, irrespective of the recording condition (*with* or *without* commutator; the same 50 pixels were analysed in both conditions; Fig. 22E). In fact, when analysing all 202,500 pixels in the FOV over 12,000 recorded frames, none of them showed an indication of failure in any of the frames in either condition, whereby a drop below 70% of the over-time average signal amplitude of each pixel was used as an indicator of putative pixel failure (Fig. 22F). Overall, the Open-MAC commutator did not affect data quality when used with Open-Ephys/ Intan^TM^ or UCLA-miniscope systems, respectively.

## Ethics statements

Validation data has been obtained from animal experiments that were approved by the Regierungspräsidium Tübingen (licence numbers TV1399 and TV1564) and that complied with the ARRIVE guidelines and were carried out in accordance with the EU Directive 2010/63/EU for animal experiments and the German Animal Rights Law.

## Declaration of Competing Interest

The authors declare that they have no known competing financial interests or personal relationships that could have appeared to influence the work reported in this paper.
