# Supplementary material for: Open-MAC: A low-cost open-source motorized commutator for electro- and opto-physiological recordings in freely moving rodents
Source: HardwareX. 2023 May 16;14:e00429. doi: 10.1016/j.ohx.2023.e00429 (PMC10209885; doi:10.1016/j.ohx.2023.e00429)
Supplement: Supplementary data 9 [file mmc9.pdf]

```
int dirPin  = A7;  // Direction fro stepper motor
int stepPin = A8; // Step for stepper motor
int EnPin   = A9; // Enable for stepper motor
//int TTL     = A0; // ADC out (can be used as TTL)
// Define Hall sensor connections
#define HALL_SENSOR_A     A1 // Hall sensor A
#define HALL_SENSOR_B     A2 // Hall sensor B
int sensorReading_delay   = 0;
int step_delay_dur = 300;
int stepsPerRevolution = 50;

void setup() {
//  Serial.begin(9600); // to debug and serial communication
  // initial speed and the target position
  pinMode(EnPin,OUTPUT);
  pinMode(dirPin, OUTPUT);
  pinMode(stepPin, OUTPUT);
  // Hall sensors
  pinMode(HALL_SENSOR_A, INPUT);
  pinMode(HALL_SENSOR_B, INPUT);
  attachInterrupt(digitalPinToInterrupt(HALL_SENSOR_A), move_backward, LOW);
  attachInterrupt(digitalPinToInterrupt(HALL_SENSOR_B), move_forward, LOW);
}

void move_forward(){
  digitalWrite(dirPin, LOW);
  // Spin the stepper motor 1 revolution quickly:
  for (int i = 0; i < stepsPerRevolution; i++) {
    // These four lines result in 1 step:
    digitalWrite(stepPin, HIGH);
    delayMicroseconds(step_delay_dur);
    digitalWrite(stepPin, LOW);
    delayMicroseconds(step_delay_dur);
  }
}

void move_backward(){
  digitalWrite(dirPin, HIGH);
  // Spin the stepper motor 1 revolution quickly:
  for (int i = 0; i < stepsPerRevolution; i++) {
    // These four lines result in 1 step:
    digitalWrite(stepPin, HIGH);
    delayMicroseconds(step_delay_dur);
    digitalWrite(stepPin, LOW);
    delayMicroseconds(step_delay_dur);
  }
}

void loop() {
  delayMicroseconds(sensorReading_delay);
 }
```
